# Lyme disease and the pursuit of a clinical cure

**DOI:** 10.3389/fmed.2023.1183344

**Published:** 2023-05-24

**Authors:** Heather Adkison, Monica E. Embers

**Affiliations:** Division of Immunology, Tulane National Primate Research Center, Tulane University Health Sciences, Covington, LA, United States

**Keywords:** Lyme, borreliosis, persistence, PTLDS, PTLD, antibiotic, autoimmune, inflammation

## Abstract

Lyme disease, caused by the spirochete *Borrelia burgdorferi*, is the most common vector-borne illness in the United States. Many aspects of the disease are still topics of controversy within the scientific and medical communities. One particular point of debate is the etiology behind antibiotic treatment failure of a significant portion (10–30%) of Lyme disease patients. The condition in which patients with Lyme disease continue to experience a variety of symptoms months to years after the recommended antibiotic treatment is most recently referred to in the literature as post treatment Lyme disease syndrome (PTLDS) or just simply post treatment Lyme disease (PTLD). The most commonly proposed mechanisms behind treatment failure include host autoimmune responses, long-term sequelae from the initial *Borrelia* infection, and persistence of the spirochete. The aims of this review will focus on the *in vitro, in vivo*, and clinical evidence that either validates or challenges these mechanisms, particularly with regard to the role of the immune response in disease and resolution of the infection. Next generation treatments and research into identifying biomarkers to predict treatment responses and outcomes for Lyme disease patients are also discussed. It is essential that definitions and guidelines for Lyme disease evolve with the research to translate diagnostic and therapeutic advances to patient care.

## 1. Introduction

Lyme disease (LD) is the most common vector-borne disease in the United States and displays an increasing trend in recent years. A recent study estimated that there have been about 476,000 new cases every year since 2010 (1) while a previous similar analysis for 2005–2010 estimated about 329,000 LD cases per year (2). LD is a bacterial infection caused by a spirochete belonging to the *Borrelia burgdoferi sensu lato* complex. In North America this infection is most commonly referred to as Lyme Disease, whereas in Europe and other locations of the world it is commonly called either Lyme Borreliosis or Borreliosis. LD is a zoonotic infection transmitted to humans through the bite of an Ixodid tick. The species of *Borrelia* and *Ixodes* involved varies by geographical region throughout North America, Europe, and parts of Asia. In North America, *Borrelia burgdorferi sensu stricto* (Bb) causes the majority of infections and is transmitted either by *Ixodes scapularis* or *I. pacificus* (3). In Europe and parts of Asia borreliosis is transmitted by *I. ricinus* ticks predominantly infected by either *B. afzelii, B. garinii, or B. bavariensis* (4). The predominant reservoir in the United States is the white-footed mouse where typically larval Ixodes ticks acquire the spirochetes via a blood meal (5). Once infected, a tick remains infectious throughout the remainder of its life cycle. Both nymphal and adult ticks can transmit the disease to humans, but it is the former that is responsible for the majority of human cases. An important aspect of LD research is understanding the complexities of how the spirochete adapts and survives the harsh tick environment, as well as the phenotypic changes required to persist in both its reservoir host and humans.

Untreated LD in humans can be divided into three disease stage progressions that include both unique and overlapping clinical manifestations. The stages are labeled as early localized, early disseminated, and late disseminated, and the various signs and symptoms include erythema migrans (EM), malaise, arthralgia, fatigue, arthritis, carditis, and various neurological manifestations such as facial palsy (6, 7). The diagnosis of LD is not as straightforward as with many other infectious diseases, where detection by culture, PCR, or serology is typically more reliable and conclusive. Laboratory diagnostic testing for LD is based upon indirect methods using a serological two-tiered testing of enzyme immunoassay (EIA) and immunoblotting with varying degrees of sensitivities based upon the stage of illness; whereas direct detection via culture or PCR is used mainly only in research studies (8–10). The issues with LD diagnostic testing can lead to false negative results and misdiagnoses that ultimately delay proper treatment. The course, duration, and success of antibiotic therapy for LD varies according to the disease stage and site of disease manifestations (joint, heart, nervous system, etc.); however, the typical treatment regimen consists of either oral doxycycline, amoxicillin, cefuroxime, or IV ceftriaxone (11, 12).

**Figure 1:**
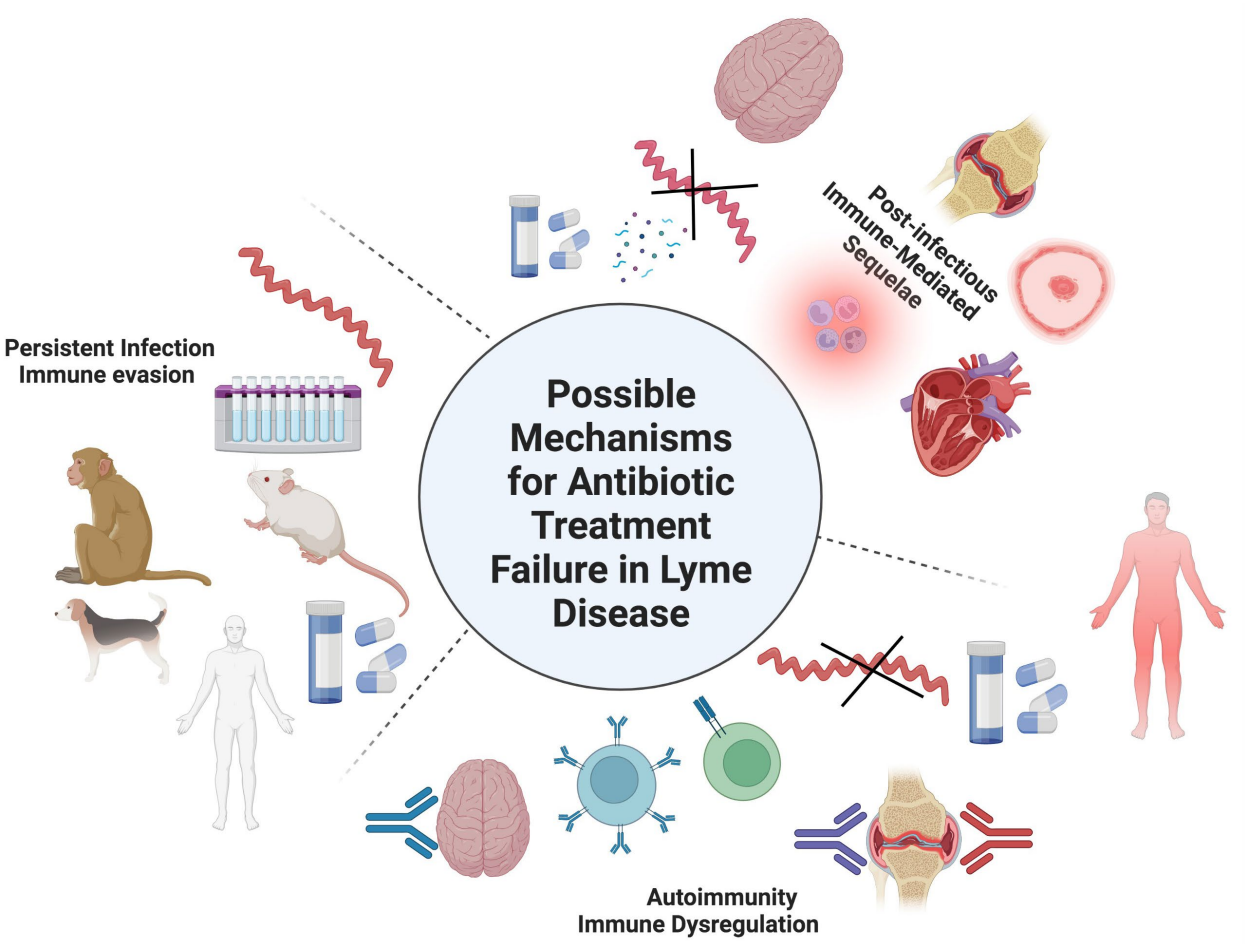
Possible mechanisms for persistent symptoms after antibiotic treatment for LD. 1) Autoimmunity in which self-antigens are targeted by B- and/or T-cells due to molecular mimicry or other immune dysregulated mechanisms. 2) Post-infectious immune mediated sequalae from the spirochete or its cellular components such as peptidoglycan could cause damage to tissues/organs during the initial infection. Evidence for these first two mechanisms doesn’t involve the recovery of whole, intact spirochetes after treatment. 3) Persistent infection in which a small number of spirochetes survive after antibiotic treatment as viable, viable non-cultivable (VBNC), or non-viable cells. Evidence for B. burgdorferi antibiotic persistence can be found in in vitro culture studies and several in vivo animal models such as mice, dogs, and non-human primates (NHPs). It should be notes that these mechanisms may not exist independently of one another and involve other unknown mechanisms.

Despite differences in treatment regimens and other methodologies, several early studies reported detection of spirochetes or their components in patients treated for LD (13–16). Further insight has revealed antibiotic therapy is most successful in cases diagnosed in the earlier stages of illness and delays in diagnosis increase the odds for treatment failure (17, 18). Retrospective studies of LD patients at least 1 year post treatment found that patients treated within 4 weeks of disease onset were more likely to have a successful treatment outcome, whereas delayed treatment led to a higher frequency (up to 50% or more) of cognitive and musculoskeletal impairment (19, 20). This was confirmed in a more recent study that demonstrated that chronic symptoms were statistically more likely to develop in LD patients where treatment was delayed for 30 days or more (21). There are three recognized outcomes with the recommended course of antibiotic therapy for LD: resolution/cure; antibiotic refractory Lyme arthritis (also referred to as post-infectious Lyme arthritis), post-treatment Lyme disease syndrome (PTLDS) or post-treatment Lyme disease (PTLD). The first two outcomes are the simplest in terms of a consensus for their definition and observations. The latter possible outcome however is wherein the most controversy resides over its definition, symptom inclusion profile, cause, and continued treatment. PTLD is estimated to affect 10–20% or of treated LD patients that present with an EM rash and is defined as persistent or relapsing symptoms such as fatigue, musculoskeletal pain, and cognitive impairment for more than 6 months after completion of the recommended antibiotic therapy (22, 23). A recent report estimated the cumulative presence of PTLD to be near 2 million people in 2020 (24). These manifestations are usually subjective and can range from mild to severely debilitating. It has been suggested that the PTLD incidence could be even higher due to the underdiagnoses of LD patients that do not present a known early-localized EM rash, and thus are not diagnosed and treated until they present with signs and symptoms of more disseminated disease (25, 26). In fact some groups have suggested alternative names for PTLD that incorporate broader definitions and inclusion criteria such as Lyme-multiple systemic infectious disease syndrome (MSIDS) (27), or by delineating chronic LD into untreated (CLD-U) and treated (CLD-PT) categories (26). This review will use PTLD to refer to persistent symptoms with varying degrees of severity following treatment for LD. A group of researchers have also attempted address the symptom heterogeneity of PTLD by dividing it into three separate subgroups based upon the severity of six different symptom profiles (28). Despite what terminology, definition, and symptom profile is used to describe this significant cohort of LD patients, they all share an element of antibiotic treatment failure. Thus, the main problem that needs solving is determining the mechanisms behind the development and pathology of PTLD. One consensus behind PTLD is the agreement for the identification of several risk factors for its development such as microbiological factors including genospecies/strain of the *Borrelia* involved or co-infection with other tickborne pathogens; host factors such as genetics, environment, and the presence of comorbidities; and clinical factors such as the previously discussed delay in proper treatment. The mechanisms and treatment for PTLD continue to perplex and create controversy among the scientific and medical communities. Several mechanisms have been suggested to play a role in PTLD, but the most common include autoimmunity, sequelae of previous active infection, and persistent *Borrelia* infection (Figure 1).

Each suggested mechanism will be discussed separately; however, it is important to note that the role these mechanisms play could exist singly, in combination with each other, or involve other unknown mechanisms. The main argument centered around treatment of PTLD concerns whether or not further antibiotic therapy is helpful, hurtful, or benign to patient care (29, 30). This review will examine *in vitro*, *in vivo*, and clinical studies that support or challenge each of these mechanisms, viewed through how the host immune system responds to infection. We also discuss research into next generation treatments and the discovery of biomarkers to predict treatment responses and outcomes.

## 2. Potential causes of antibiotic treatment failure for Lyme disease

### 2.1. Autoimmunity

The purpose of the immune system is to combat and prevent infections. Its ability to perform that duty is critically reliant on being able to distinguish self from non-self. Autoimmune disorders occur when that line gets distorted and results in an immunological assault on self-tissues. The pathology of several autoimmune diseases such as Systemic Lupus Erythematosus (SLE), Myasthenia Gravis, and Grave’s disease in which self-reactive T-cells and/or antibodies attack host tissues are well-characterized (31–33). The etiology of most autoimmune disorders is thought to arise from a mixture of genetic and environmental factors. However, the role of specific microbial antigens in triggering autoimmune disorders is less well understood. Perhaps the most well-known association between a specific infectious agent and autoimmunity is between the gram-positive bacteria *Streptococcus pyogenes* and rheumatic fever. Yet there are several autoimmune and chronic inflammatory conditions in which microbial antigens are thought to trigger the self-reactive response, including Crohn’s disease, ulcerative colitis, Multiple Sclerosis (MS), and Guillain-Barre’ syndrome (GBS) (34–36); however, the traditional Koch’s postulates are not sufficient to determine the complex etiology for these diseases (37). It is understood that the presence of autoreactive T-and/or B-cells alone is not enough to induce an autoimmune reaction. A breakdown in central and peripheral tolerance must also happen before an autoimmune disorder can manifest (38). There are several mechanisms of how autoimmunity induced by foreign antigens can occur such as molecular mimicry, epitope spreading, bystander activation, and polyclonal B-and T-cell activation due to persistent pathogens and chronic inflammation (39). Molecular mimicry, the most investigated autoimmune mechanism regarding LD, refers to the idea that a high degree of similarity between *Borrelia* antigens and self-antigens can lead to a cross reactivity of T cells and/or antibodies to host tissues. The most evidence and support for molecular mimicry in PTLD originates from research in Lyme arthritis. The so-called “post-infectious Lyme arthritis” is one of the more well-characterized possible outcomes after antibiotic treatment, but it is generally considered to be clinically distinct from PTLD due to a lack of other clinical manifestations such as fatigue and malaise.

In the early 1990s, several researchers investigating the role of autoimmunity in association with chronic LD symptoms found homology between a few *Borrelia* proteins and human proteins (40–42). The correlation between autoimmunity and antibiotic refractory Lyme arthritis was originally supported by its association with specific MHC II alleles, such as HLA-DRB1 (43, 44). Further investigation into treatment of refractory Lyme arthritis also revealed a connection between HLA-DR4 alleles and antibody to *B. burgdorferi* (Bb) outer surface lipoproteins OspA and OspB (45). Thus, investigations into the hypothesis that molecular mimicry between specific *Borrelia* proteins and human proteins was the autoimmune source behind treatment resistant Lyme arthritis began. Further research found sequence homologies between human leukocyte function-associated antigen-1 (hLFA-1 α) and Bb OspA (46, 47). Autoreactive T cells and/or B cells to a variety of human proteins such as the heat shock protein (hsp) family, myosin, myelin basic protein (MBP), hLFA-1, annexin A2, endothelial growth cell factor (EGCF), apolipoprotein B-100 (apoB-100), and matrix metalloproteinsase-10 (MMP-10) have been correlated with post treatment Lyme arthritis (41, 46, 48–51). However, a study identifying several cross-reactive self-antigens as ligands that also recognize OspA-specific T cells demonstrated that cross-reactivity may be a common occurrence and that its existence alone does not necessarily imply that it will result in autoimmune pathology (52). Thus, molecular mimicry between spirochetal antigens and host proteins alone is likely not enough to induce persistent Lyme arthritis. This is supported by a mouse study that determined both CD4+ and CD8+ T cells were stimulated via bystander activation through an upregulation of TLR2 expression on T cells (53).

Further evidence of a link between an autoimmune mechanism and antibiotic refractory Lyme arthritis involves the role of regulatory T (T_reg_) cells. Lyme arthritis patients with lower levels of T_reg_ cells in synovial fluid had longer durations of symptoms in antibiotic refractory Lyme arthritis (54), and a subsequent study using a mouse model demonstrated that T_reg_ cells may play an anti-inflammatory role to limit arthritis development with *B. burgdorferi* infection (55). In addition, a TH17 immune response in the early phases of the later stages of LD correspond with development of autoantibodies and antibiotic refractory Lyme arthritis (56). Another possible mechanism could involve B cells and the phenomenon of epitope spreading and post-translational or epigenetic modification of self-proteins, which has been seen in rheumatoid arthritis (57). These studies imply that disruption to immune regulation in conjunction with the presence of autoreactive B and/or T cells likely play a role in the pathology behind antibiotic refractory Lyme arthritis. The success of reducing or eliminating symptoms when treating antibiotic refractory Lyme arthritis with immune-modulating drugs such as methotrexate and TNF inhibitors strengthens this role of this mechanism (58).

However, when these same autoimmune hypotheses and research are applied to other Lyme disease pathologies and conditions such as Lyme carditis, Lyme neuroborreliosis, and PTLD, the results and conclusions are variable. In the late 1990s and early 2000s a group of researchers identified several Bb and human protein sequences that matched a T cell clone generated from the CNS of a Lyme disease patient; however, during *in vitro* testing, the clone recognized and bound human proteins (autoantigens) at much higher concentrations compared to the *Borrelia* proteins, emphasizing that autoimmunity from molecular mimicry may only happen when very specific conditions are met (59, 60). This finding is consistent with the idea previously mentioned that the presence of self-antigens may be a fairly common occurrence and these autoreactive T or B cells may not necessarily depend on the presence of Bb antigens to develop. An *in vivo* study using mice showed evidence of molecular mimicry between murine myosin protein and Bb OspA to aid in generating a cross-reactive response against heart and musculoskeletal tissue, as well as finding that in the presence of autoimmune conditions, Bb caused more inflammation and persisted longer in mouse heart tissue (61). Various host factors and genetic predispositions to autoimmune disorders may play a role in the severity of Lyme carditis as well as other Lyme disease pathologies.

A group of researchers studying autoantibodies in Lyme arthritis actually found that a small number of patients with carditis in the early disseminated stages of LD had increased levels of IgG3 autoantibodies to apoB-100 compared to patients with neuroborreliosis or arthritis (62). However, how these patients responded to antibiotic treatment was not part of the study’s design. Thus, whether or not apoB-100 or myosin autoantibodies continue to be elevated and contribute to the pathology behind carditis or even PTLD remains to be investigated. The role of TH17 cells and autoimmunity in PTLD is also unclear. A study using IL-23 levels to measure the TH17 immune response in PTLD patients compared to treated LD patients without symptoms found that the PTLD group had a statistically significant increase in IL-23 levels and were commonly correlated with autoantibodies to EGCF that, however, did not display any statistical significance (63).

Since the clinical manifestations of PTLD are mainly subjective and many likely involve the central and peripheral nervous systems, an autoimmune response against neural tissues is a logical hypothesis and could provide a link between Lyme neuroborreliosis and PTLD. Antibodies against assorted neural proteins were found to be significantly higher in PTLD patients vs. post-Lyme healthy controls and would seem to support an autoimmune mechanism for chronic neurological Lyme disease symptoms (64–66). However, seronegative PTLD patients also had anti-neural antibodies, suggesting additional antigen-independent mechanisms likely also play a role in the development of PTLD (64). Since molecular mimicry is antigen-dependent, perhaps other autoimmune mechanisms could be at play, such as epitope spreading or bystander activation, a likely culprit behind antibiotic refractory Lyme arthritis. Whether or not the correlation between PTLD and the presence of these anti-neural antibodies could be the pathological cause for the neurological manifestations of PTLD needs to be further investigated. In that regard, a study in 2018 examined autoantibodies against a glycolytic enzyme, gamma enolase, from a LD patient as a possible cause for the chronic neurological symptoms in some cases of PTLD by interfering with glycolysis in nerve cells (67). A previous study also suggested a possible role for the cytokine IFN-alpha behind the immune dysregulation mechanism for persistent neurological symptoms (66).

Unlike the connection between Lyme arthritis and specific HLA-DRB alleles, as association between PTLD patients and a specific HLA class II genotype has yet to be identified that would strengthen the argument for an autoimmune pathology (68). Though there are very few such studies in the literature specifically related to PTLD and host genetic factors, this does not rule out innate or adaptive immune system genetic elements in the generation of the dysregulated immune response. Immunological responses, other than autoimmunity, and microbial factors during early and disseminated LD are likely to have plausible roles in PTLD pathogenesis.

### 2.2. Post-infectious immune-mediated sequelae

Clinical manifestations experienced during an infection are either the result of the direct action of the infectious agent and/or its toxins, or the indirect product of the host immune response to the infection. These reactions usually subside and resolve after the infection is eliminated, homeostatic conditions are restored, and the host repairs any direct or collateral damage. This section will examine the literature involving the innate and adaptive immune responses in early and disseminated LD and how their role is possibly linked to the pathogenesis of PTLD. Bb infects its reservoir hosts persistently without causing any disease pathology such as arthritis or carditis, and the spirochete also lacks any known endo-or exotoxins in its genome. Thus, the clinical manifestations of LD in its non-reservoir hosts, such as humans, are due to the immune response to the pathogen (69, 70). These immune-mediated effects are directed by cells of the innate and adaptive immune system through the complex interactions of various pro-and anti-inflammatory chemokines and cytokines. In connection with this hypothesis as a potential mechanism for PTLD, a study published in 2015 that followed antibiotic treated patients with culture-confirmed early LD for 10–20 years observed not only that the nonspecific symptoms of PTLD can persist for extremely long periods of time, but also the symptoms for a very small subset of patients they followed could possibly have been due to nerve damage from the initial LD infection (71). PTLD studied in patients who developed EM during the early phase of infection showed that the average duration of acute illness was significantly longer in patients developing persistent than in patients with resolved illness (72). In addition, a recent study on carditis in a murine model demonstrated changes in the regulation of mitochondrial components of the heart tissue as well as functional changes in macrophages as a consequence of infection with *B. burgdorferi* (73). This study, however, did not examine the effect of antibiotic treatment in the infected mice, but does support the importance of a timely diagnosis and treatment before potential long-term, chronic damage can be generated., Functional changes to mitochondrial components in other tissues besides the heart, such as nerve and skeletal muscle that are often targeted during Bb infection, remains to be investigated. Such research could provide insight into a few of the chief symptoms among PTLD patients including fatigue, cognitive changes, and musculoskeletal pain or weakness. When Bb affects the nervous system, neuroborreliosis, the cascade of immunomodulatory inflammation can trigger apoptosis of glial cells leading to an assortment of neurological manifestations (74–76). If the majority of this damage is sustained before antibiotic treatment is initiated, then it is conceivable that some of the symptomology could continue during and after antibiotic treatment considering the amount of time it takes the body to repair nerve cells (77). However, how some patients who suffer PTLD symptoms for many years fit into this explanation is unclear.

Two of the larger questions at hand are how could differences in the immune response to Bb infection contribute to persistence of symptoms after antibiotic treatment and why do they occur? To analyze these questions, a brief understanding of the pathogenesis and immune response to Bb is required. There are several excellent reviews that give a detailed and thorough understanding of the immune response and pathogenesis of LD in both animals and humans such as those by Coburn et al., Radolf, and Bockenstedt (69, 70, 78). Both the innate and adaptive immune responses are important for controlling the spirochetal burden in various tissues. The initial innate immune response is mainly triggered via the surface pathogen recognition receptor TLR-2 signaling leading to NF-kB and an inflammatory cytokine response. This response is critical to controlling the bacterial burden of the initial infection by recruiting macrophages, neutrophils, and other lymphocytes to the site of infection. It also appears that interferon gamma (IFN-ɣ) is a major instigator in the inflammatory pathology seen in early LD, especially in the joints and nervous system (79–82). IFN-ɣ from macrophages stimulated *in vitro* by Bb outer surface proteins increased the production of nitric oxide (NO) as well as other pro-inflammatory cytokines that contribute to the inflammatory pathology seen in LD (83). Consequently, tight regulation of these inflammatory components is needed during infection to minimize damage to host cells and tissues and allow a return to homeostatic conditions. The expression of the Th1 cytokine IFN-ɣ was decreased in the skin of European patients with persistent symptoms after being treated for EM compared to post-treatment asymptomatic patients, who exhibited a more prominent IFN-ɣ response (84). Other groups studying the differentiation between Th1 vs. Th2 immune responses in patients with antibiotic refractory Lyme arthritis observed that a robust IFN-ɣ response correlated with a decrease in the expression of genes for tissue repair, proliferation, and metabolism (85, 86).

Inflammation of the CNS has been found in mice during early Bb infection and induces changes histologically and in the expression profiles of inflammatory genes, especially interferon-stimulated genes (ISGs), which can also be induced when no spirochetes can be detected in the brain parenchyma (82). The differences between these IFN-gamma studies further support claims that the role of specific immune signals for manifestations of LD may be unique according to the system affected (87–89). Therefore, it further maintains the observations that antibiotic refractory Lyme arthritis and PTLD are distinct disease states with unique pathologies and triggers. Additional cytokines and chemokines also are important in the response to Bb infection and the pathology for PTLD. Another TH1-associated chemokine, CCL19, was shown to be elevated in some PTLD patients compared to patients who remained asymptomatic after treatment, suggesting possible ongoing immune-mediated reactions after treatment (90). Study of IL-10 levels in mouse models found a correlation between higher levels of IL-10 with decreased levels of TNF-α, suggesting that circumstances which lead to less IL-10 production during Bb infection could result in more TNF-alpha mediated inflammatory damage to various tissues (91).

While the innate immune response during early infection is indispensable for controlling the bacterial burden, it is the adaptive immune system that becomes crucial during the later disseminated stages of infection. Studies using severe combined immune-deficient (SCID) mice infected with Bb show that initially they are able to control the bacterial burden the same as the wild-type mice; however, after several weeks the inflammation remained active in the SCID mice while it was fairly resolved in the immunocompetent mice (92–94). It has also been shown that a strong B-cell/antibody response to Bb in treated patients led to a quicker return to health than patients that had a poorer B-cell response (95). However, the antibodies generated during *Borrelia* infection must be able to penetrate and access the tissues, such as the joints and heart, in order to help ameliorate the inflammation (70). It is also clear that T-cells also have a more marked role in the pathology in human LD compared to numerous strains of inbred laboratory mice, and this is evident in the immune cell composition of skin, joints, heart, and cerebrospinal fluid (CSF) of human patients infected with Bb (70). CD4+ T cells appear to help generate antibody production during LD, but do not aid in affinity maturation or class switching to provide long-term protection (96). These findings reinforce the notion that a strong antibody response is central to controlling the infection, but it is not able to provide long-term protection in the form of memory cells. Regulatory T cells (Tregs) are necessary for controlling the amount of inflammation and tissue damage during Bb infection. The Th17 cell-associated cytokine IL-23 has also been found to be increased among certain cohorts of PTLD patients (63).

Another important consideration in the differential immune response is the form/shape of the spirochete during the course of infection. The induction of cell-wall deficient, round bodied, and biofilm-like forms of Bb *in vitro* has led to the interrogation of the infectivity and the pathogenicity of these forms *in vivo*. An *in vitro* analysis showed that the round body form of Bb showed differential phagocytosis by macrophages, cytokine responses, as well as protein and antigenicity profiles than the traditional spirochetal form of Bb (97). These different morphological forms of Bb are more difficult to study under host adapted conditions, therefore, their contribution and significance to the pathology behind LD and PTLD in humans is not clear. A recent report described the antibiotic tolerance and pathogenicity of biofilm-like microcolony (MC) forms, stationary phase (SP) forms with round bodies and logarithmic phase (LOG) forms of Bb. MC and SP variant forms were not only more tolerant to the standard antibiotics used for Lyme, but also caused more severe arthritis in mice than the log phase spirochete form (98).

Ongoing immune responses to bacterial components such as nucleic acids, peptidoglycan, bacterial lipoprotein debris, and outer membrane vesicles are also plausible sources for inflammatory responses in PTLD. Bb DNA has been discovered in infected mice after antibiotic treatment (99), and *in vitro* cultures of Bb treated with ceftriaxone revealed that DNA could be detected up to 56 days after treatment (100). The residual spirochete DNA is also capable of triggering recognition by PRRs of immune cells and a subsequent inflammatory response in mice suggesting that some of the symptoms after antibiotic treatment could be explained by persistent material from the original infection (101). Peptidoglycan from Bb, which does elicit a pro-inflammatory response in human PBMCs, has also been shown to persist in patients with Lyme arthritis (102). What contribution this has towards the development of PTLD is unclear, especially in cases where symptoms persist for years.

Some of the research studies into the pathology of immune-mediated sequelae, separate from autoimmunity, do not characteristically take into consideration the duration of the effects and/or any antibiotic treatment. Care should also be taken in comparing immune responses between reservoir hosts, different strains of inbred laboratory mice, and non-reservoir hosts such as nonhuman primates (NHPs) and humans. In order to ascertain the etiology of tissue or nerve damage found months and years after the completion of antibiotic treatment, two significant factors would need to be addressed: (1) re-infection and/or (2) persistent infection. While laboratory studies can easily control for re-infection with Bb, human and veterinary case studies, especially those in highly endemic regions for LD, would benefit from conclusive diagnostic markers that could differentiate between recent and past *Borrelia* infections.

### 2.3. Persistent infection

In the absence of any therapeutic intervention, *B. burgdorferi* establishes a persistent/chronic infection in both its reservoir and non-reservoir hosts. This aspect of Lyme disease is not contentious, and there is a great deal of information supporting the immune evasion tactics of the spirochetes, especially in regard to gene regulation and protein expression that enables the spirochete to survive some fairly unfavorable and dynamic conditions in its arthropod and mammalian hosts (6, 103). For example, spirochetal burden in immunocompetent mice infected with Bb peaks about 2 weeks after infection and then its numbers decline, but still is found to persist even up to a year after infection (104). Similar observations have been found in untreated dogs, horses, and NHPs infected with *B. burgdorferi* (105–107). The disagreements concerning chronic infection with Bb revolve around the question of whether or not the bacteria can persist after antibiotic treatment and whether or not they are capable of causing the symptoms experienced in PTLD patients.

In order to address the topic of persistent infection, the definition and parameters of what bacterial persistence is needs to be acknowledged. The term “persisters” was first coined by Joseph Bigger in the 1940s to describe the small fraction of bacterial cells that survived penicillin treatment by entering a nondividing state and whose progeny did not demonstrate any antibiotic resistance (108). Since then, there has been a great deal of research into antibiotic tolerance and its mechanisms for a diverse range of bacterial genera and its role in chronic infections. There are many bacterial species associated with persistent infections in humans such as *Mycobacterium tuberculosis*, *Pseudomonas aeruginosa*, *Escherichia coli*, *Staphylococcus aureus*, and *B. burgdorferi* (109). Bacterial tolerance is very different from resistance in that the bacteria are not actively growing in the presence of the antibiotic and there is no heritable genetic change in the persistent bacteria’s genome (110, 111). The stress response and other mechanisms allow bacteria, including *Borrelia*, to survive lethal conditions such as limited nutrients, extreme pH levels, and the presence of certain antibiotics. A thorough review for understanding persister cells and how they are studied *in vitro* can be found in Balaban et al. (112). Essentially, drug-induced bacterial persisters demonstrate a biphasic killing curve and the regrown persister cells have the same minimum inhibitory concentration (MIC) to the administered antibiotic as the original population but a higher minimum bactericidal concentration (MBC) (112).

#### 2.3.1. Antibiotic tolerance by *Borrelia burgdorferi*

Several early reports linked LD patients with ongoing symptoms treated with different regimens of antibiotics to a persistent *Borrelia* infection (13, 113, 114). This sparked the investigation for surviving spirochetes both *in vitro* and *in vivo* using animal models. The growth requirements, slow growth rate, genetic composition of *B. burgdorferi*, and the occult nature of the infection brings challenges to studying persistence both *in vitro* and *in vivo*. Despite these challenges, several independent studies have demonstrated that Bb can form drug-tolerant persister cell *in vitro*. A preliminary *in vitro* study aimed at examining the viability of two different Bb strains showed the inability to re-culture spirochetes from ceftriaxone-treated mice but the detection of nucleic acid (100). Interestingly, the same study also displayed a variability in the rate in the declining viability between the two Bb strains (100). In contrast, time-kill assays using a variety of antibiotics, including ceftriaxone, confirmed a biphasic killing curve of Bb and that the regrowth of surviving cells were persisters based upon MIC values (115). In this study Bb formed persisters against all the drugs tested but one, Mitomycin C. Another study determined that the development of persister cells are likely driven by a slower growth rate, demonstrating that Bb treated with doxycycline during the stationary growth phase had a higher probability of regrowth after removal of the antibiotic compared to those in the exponential growth phase (116). This phenomena in stationary phase treated cultures was also reported by Sharma et al., and both independent findings support a previous report that Bb, which has a much slower replication rate, developed persister cells *in vitro* at higher frequencies than *E. coli*, which has a much faster doubling time (117). This phenomenon is also seen in slower growing strains of other bacteria (118). Collectively, these results demonstrate the capability of Bb to develop persister cells *in vitro* and support a mechanism of persistence that has been observed in various animal models after antibiotic treatment.

*In vivo* studies of Bb antibiotic persistence often use different parameters that can make comparisons between two or more studies difficult: (a) variability in the strain and growth phase of Bb used for infection; (b) the choice of the animal model, in particular the inbred strain of the murine model; (c) the mode of infection (needle-inoculated vs. tick); (d) the timing, choice, duration, dose, and delivery of antibiotic in treatment; (e) the amount of time allowed to elapse between the end of treatment and necropsy; and (f) the methods used to detect the spirochete such as culture, DNA/RNA, xenodiagnoses, and immunofluorescence. Several different animal models have been used to study and confirm antibiotic persistent *Borrelia* infection such as dogs (106, 119, 120), horses (105), mice (98, 121–125), and non-human primates (NHPs) (107, 126, 127). The conclusions for persistence are based upon the ability to culture Bb from either tissues or xenodiagnostic ticks, detect nucleic acid (DNA and/or RNA) to various Bb housekeeping and outer surface protein-encoding genes from both tissues and xenodiagnostic ticks, and/or immunofluorescence (IFA) of Bb antigens in various tissues from infected and treated animals. Immunofluorescence staining was able to detect, albeit rarely, morphologically intact spirochetes in the brain or heart of three individual NHPs that had been treated with doxycycline for 28 days (127). Xenodiagnosis involves placing uninfected *Ixodes* ticks and allowing them to feed on the host animal then examining the fed tick for the presence of Bb through culture, PCR, and/or IFA. While this has been useful for several murine and NHP studies mentioned previously, its use in humans has had very limited success (128).

Some studies claim that Bb does not survive antibiotic treatments because of the failure to re-culture the organisms shortly after antibiotic treatment has ended (129, 130), while sometimes only nucleic acids or antigens from Bb are detected in various treatment models (99–101, 131). The discrepancy between these two vastly different conclusions regarding antibiotic persistent infection is perhaps one of the more contentious topics for mechanisms behind PTLD among clinicians and researchers. Studying persistent Bb infection in animals and humans involves distinguishing between viable, viable-non cultivable (VBNC), and/or non-viable *Borrelia* and their potential role in the pathogenicity of PTLD. It has been recommended that these heterogenous descriptions of persister cells be addressed in an updated version to Bigger’s original “persisters” definition (132). Several researchers assert that VBNC and non-viable *Borrelia* that remain after antibiotic treatment are not the cause behind the symptoms for PTLD (133–135). In contrast to these claims, a group of researchers revealed that VBNC Bb located in the tissues of immunocompetent, antibiotic treated mice could cause disease when transplanted into immunocompromised mice (123). Also, various murine tissues demonstrated a resurgence of Bb DNA 12 months after antibiotic treatment comparable to DNA levels found in the untreated control group (124). Thus, some animals thought to be free of Bb when examined shortly after treatment stops may still be harboring a few persistent spirochetes that take several months to regrow to a level that can be detected on a molecular level. This later report also very importantly demonstrated via RNA transcription of various host factors that the VBNC Bb was still capable of eliciting a host response (124). The discovery of Bb peptidoglycan during post infectious phases of Lyme arthritis could also lend support to persistent infection with intact spirochetes in that only actively dividing *Borrelia* continually shed peptidoglycan into the extracellular environment (102).

Consequently, symptoms that persist for several months to years after treatment could be attributed to a response to only a very few functioning spirochetes and various components that simply are not perceived using conventional detection methods such as culture or serology. Earlier *in vitro* studies found live *Borrelia* activated human monocytes to produce more pro-inflammatory cytokines than lysates containing lipids, proteins, carbohydrates, etc. from *Borrelia* (136, 137). A recent report also found that non-viable Bb actually had a greater neuropathogenicity effect on frontal cortex and dorsal root ganglia explants than intact-viable Bb in a rhesus macaque *ex vivo* model (138). While this mainly has implications for understanding the pathogenicity of neuroborreliosis, it does demonstrate that non-viable *Borrelia* are still capable of eliciting an immune response. A study published in 2021 used IFA and PCR on post-mortem central nervous system tissues to identify Bb DNA in the brain and an intact spirochete in the spinal cord of a patient that had known history of a LD diagnosis and treatment; however, the study was unable to confirm if any spirochetes were still metabolically active or if they played any role in the neurodegenerative disease that lead to the patient’s death (139). A study in 2019 found that Bb treated with doxycycline *in vitro* displaying diminished motility were able to infect *scid* and C3H mice based upon positive cultures and/or RT-PCR from organ tissue (140). Together these findings imply that VBNC and/or non-viable Bb are not just a benign presence in incidental hosts, especially humans. More research is needed to affirm and connect these correlations and findings to biological mechanisms and pathways that could provide more specific explanations for the symptoms of PTLD.

#### 2.3.2. Antibiotic tolerance mechanisms: gene expression, morphology, and immune evasion

There are various mechanisms that allow bacteria to adapt and survive under a vast range of conditions. Some of these mechanisms include changes in gene and protein expression, changes in morphology and growth in biofilms, and immune evasion tactics. Antibiotic tolerance and the development of the previously discussed persister cells is possibly an essential mode of survival for *Borrelia* that utilizes these mechanisms. This section will discuss briefly the genetic mechanisms behind the formation of drug-tolerant Bb persister cells, as well as how morphological differences, growth changes, and immune evasion tactics likely play a role in the persistence of Bb in incidental hosts such as humans.

Exposure to various environmental stressors is known to induce the formation of bacterial persisters. These stressors include changes in pH, temperature, nutrient availability, and antibiotics. Faster growing bacteria such as *E. coli* and *Salmonella* are typically used to study the genetic mechanisms related to antibiotic tolerance and bacterial persister cell formation; nevertheless, mechanisms that induce persister cell formation in Bb are gradually becoming understood. Cabello et al. have published an extensive review of the scientific literature regarding the molecular mechanisms that trigger formation of persister cells of Bb and PTLD (141, 142). Briefly, changes in environmental conditions, such as changes in amino acid availability, temperature, pH, etc., trigger the stringent response that leads to alterations in growth, motility, transport of amino acids, utilization of different sugars, and morphology (141, 142). Researchers have examined differences in protein expression using RNA sequencing between persister cells formed during amoxicillin and doxycycline treated Bb and non-treated controls *in vitro* (143). This study found several similarities in the genes that were up-regulated and down-regulated between the two different antibiotic-treated groups. Both doxycycline and amoxicillin had two-fold increases in expression in genes corresponding to transport, DNA repair, chemotaxis, and membrane or envelope proteins, while also showing two-fold decreases in genes associated with numerous outer membrane proteins and ribosomal proteins. Additional evidence for doxycycline upregulating gene expression in genes involved in persister cell formation can be found in a study using next generation RNA sequencing on doxycycline treated Bb, treated Bb after regrowth, and untreated Bb (140). Together these results support changes in gene expression during exposure to antibiotics aids the survival and persistence of spirochetes in humans.

In addition to the common spiral shape attributed with spirochetes, *Borrelia* is actually pleomorphic, capable of transforming into atypical, non-motile shapes such as L-form, blebs, round bodies or cystic forms, and even found to grow in what can only be described as biofilm-like conditions (144–147). The same conditions, such as lack of nutrients and antibiotics, that trigger the stringent response mentioned previously are used *in vitro* to observe these morphological changes (144, 145). When deprived of amino acids, fatty acids, and lipids, serum-starved Bb rapidly changes to round or cystic forms (148). These transformed spirochetes, however, are capable of reverting back to their normal and motile shape (145, 149). Some of the genes up-regulated from exposure to amoxicillin are thought to aid in the change to the round bodied form (143). Atypical Bb identified as cystic and granular forms were found in the CNS of three patients suffering from chronic neuroborreliosis via immunohistochemistry and microscopy (150). However, based upon in-depth reviews of the scientific literature, it is not entirely clear what role morphological variants of Bb may play in PTLD (151). *Borrelia* have been demonstrated to form aggregates in ticks and under certain *in vitro* culture conditions, and this has led to the hypothesis that these biofilm-like aggregates of Bb could play a role in antibiotic treatment failure and persistent infections in humans (146, 152, 153).

Bb has many immune evasion strategies against both the innate and adaptive immune responses. There are numerous in-depth reviews that discuss these strategies at length from the beginning of the infection cycle with transmission of Bb during tick feeding all the way through local and disseminated stages of infection (103, 154, 155). This review will only focus on areas of immune evasion that are more involved with antibiotic persistence of Bb and PTLD since the commonly most prescribed antibiotics for LD, such as doxycycline and amoxicillin, are bacteriostatic drugs that target actively dividing cells and rely on the immune system to clear debris and target any slower growing spirochetes.

One very effective evasive maneuver is disseminating into tissues that immune cells are either not frequently found or cannot access. Bb is only transiently found in the bloodstream due to its relatively quick dissemination and preferences for distal body sites such as joints, heart, and brain. Thus, if phagocytic cells or large antibody molecules such as IgM cannot eliminate any potential remaining spirochetes that are able to tolerate antibiotic exposure, those remaining cells can remain in those sites for prolonged periods of time. It has also been hypothesized based upon *in vitro* studies that Bb may also avoid clearance by the immune system by residing temporarily inside various cell types such as dermal fibroblasts and chondrosarcoma cells (156, 157). However, evidence has yet to be established for intracellular infection of Bb from either *in vivo* or clinical studies.

## 3. Next generation treatments and biomarkers

Since the scientific community cannot come to a consensus for a pathological cause behind PTLD, it makes sense that treating PTLD is another source of disagreement among clinicians and researchers. There is division regarding the use of further antibiotic treatment for patients with PTLD (29, 30, 158, 159). There are several studies that conclude additional antibiotic treatment is not beneficial for persistent LD symptoms (160–162). Yet, in contrast, a biostatistical review of the literature found that retreatment of LD with antibiotics may be beneficial in certain cases (163). These discrepancies likely revolve around the complexity and lack of consensus over definitions and diagnosis guidelines for PTLD. Research for treating PTLD is typically either based around autoimmunity and other immune-mediated factors, and/or persistent infection, and most drugs fit into one of three categories: antibiotics, pathogen specific inhibitors, and immune therapies. Most studies are *in vitro* and *in vivo*, while a smaller percentage of treatments have been or are currently undergoing clinical trials.

### 3.1. Antibiotics and pathogen-specific inhibitors

New and existing antibiotics have been discovered to have potential against bacterial persister cells from various bacterial genera including *Staphylococcus* and *E. coli* that could either directly kill persisters or re-activate persister cells (164–168). Most studies using antibiotics and small molecule drugs for LD and PTLD are generally either novel synthetic drugs or homeopathic extracts that have not yet received FDA approval, or use pre-approved drugs that are repurposed alone or in combination with current antibiotics.

Several studies have screened vast drug libraries, such as those from the Food and Drug Administration (FDA) and National Institute of Health (NIH), for compounds that have activity against the various forms and growth states of *Borrelia* (169–173). Some drugs that already have FDA approval to treat other disorders and conditions, such as the drug disulfiram, have demonstrated borreliacidal activity *in vitro* and *in vivo* (174–176). Consequently, in the future several medications may be repurposed for new uses in infectious diseases. There is evidence that the antibiotic vancomycin may have efficacy against stationary phase Bb based upon cultures and evaluation in SCID mice (177). The rediscovered hygromycin A has shown efficacy against Bb, without disturbing the gut microbiome like so many broad-spectrum antibiotics, *in vitro* and in an *in vivo* mouse assay, but it’s use in preventing persistent infections was not determined (178). Various drug combinations such as daptomycin or artemisinin, cefoperazone, and doxycycline as well as the combination sulfachlorpyridazine, daptomycin, and doxycycline have shown evidence of *in vitro* activity against Bb persisters and round bodied forms (117, 172). A separate study also discovered that the antibiotics azlocillin and cefotaxime are effective in *in vitro* killing against Bb persisters induced by doxycycline, which appear to be more tolerant to other antibiotics (179). Therefore, previous treatment with doxycycline should be taken into account for future *in vivo* and clinical therapeutic LD or PTLD studies. The efficacy of antibiotics against biofilm-like colonies of Bb are not as fruitful in discovering successful single or combination drugs. Drugs such as ceftriaxone and mitomycin C have not been shown to eradicate these structures but combinations including daptomycin or daunomycin, doxycycline, and cefuroxime have had success in sterilizing *in vitro* Bb biofilm-like microcolonies (180, 181). Dapsone, used in treatment of leprosy, has had success when combined with other antibiotics in killing biofilm-like Bb (182), and dapsone has had positive effects in a small PTLD and co-infection clinical study (183).

The discovery and development of new drugs against Bb is a much more expensive and slower process. Essential oils such as those from oregano, cinnamon bark, clove, and various flowers, grasses, and berries, as well as natural compounds such as those found in bee venom and its component melittin have demonstrated potential *in vitro* growth inhibition against Bb and its various forms (184–188). However, these discoveries need further refinement to identify specific chemical compositions and activity of these compounds as well as safety and pharmacokinetic studies in several animal models before a more conclusive determination for their efficacy can be made. Other novel treatments recommended for targeting Bb include inhibiting colonization by blocking binding to glycosaminoglycan (GAG) proteins (189), delivering toxic molecules and binding essential enzymes (190) and bacteriophage therapy (191). These innovative ideas are still in early phases of development and testing.

### 3.2. Immune therapies: biologicals and immune-modulating adjunctive treatments

Since the host immune response is central to generating and resolving the signs and symptoms of LD, the last category of therapeutics encompasses the use of antibodies and immunomodulating drugs. Immunomodulatory drugs affect the immune system by either dampening or enhancing the immune response. Potential therapeutic targets that could modulate innate and/or adaptive immune response towards Bb could be used in conjunction with antibiotic treatment in hopes of preventing chronic infection (192, 193). Unfortunately, studies testing immunomodulatory drugs for treating LD or PTLD are not widely available yet in the literature. However, modulating the level of neutrophil chemoattractants during early LD infection may help to attenuate infection and the development of Lyme arthritis and carditis (194, 195). Curative treatment during early LD stages is a key to solving the current PTLD public health problem. In addition to modulating the immune response using small molecules and compounds, therapeutic monoclonal antibodies (mAbs) also called biological therapies could be effective in providing a clinical cure for LD and PTLD. Several types of cancers, autoimmune, and infectious diseases use mAbs to target soluble cytokines or membrane-bound proteins and can even be conjugated with small molecule drugs for efficient targeting and delivery (196). There are only a few approved mAbs approved for treating bacterial infections such as those caused by *Clostridium difficile* or *Bacillus anthracis* (197), and currently there are no approved biological drugs for PTLD treatment. There is, however, current research into the use of humanized mAb to OspA for preventing transmission of *Borrelia* in both mice and NHPs (198–200). Antibody derived from anti-*Borrelia* plasmablasts could be an achievable next generation treatment (95). Also, there are hopes for future therapeutic development and evaluation combining immune therapies, such as mAbs, with antibiotics to target Bb persistence associated antigens (140).

### 3.3. Biomarkers

Biomarkers can fall into several categories such as diagnostic, prognostic, and treatment response markers. Identification of biomarkers for various stages of LD (localized vs. disseminated) and PTLD would be highly beneficial to improve the identification, prognosis, and evaluation of therapeutics. A preliminary study found 692 distinct proteins in the CSF of PTLD patients differed from other protein signatures found in patients with chronic fatigue syndrome (201). This could help distinguish PTLD patients from other post-infectious syndromes that manifest similar symptoms such as fatigue and musculoskeletal pain. Conversely, two European studies found that PTLD patients did not significantly differ in laboratory or clinical characteristics from similar symptomatic cohorts, but did discover several significant proteins in patients with neuroborreliosis (202, 203) Reasons for these discrepancies could include differences in Bb strains according to geography, and the research strategy design such as size and inclusion criteria. Given that genetic background can affect the immune response, researching genetic polymorphisms among LD patients to determine predictive and treatment response biomarkers seems a logical step. A study in Europe evaluated levels of pro-inflammatory cytokines and single nucleotide polymorphisms (SNPs) among different categories of borreliosis patients and found increases in IL-1β and IL-8, decreases in M2 macrophages, and a shift in the IL-6 promoter SNP rs1800795 in patients persistently infected compared to other groups and healthy controls (204).

The IgG antibody to the C6 Bb peptide is a useful diagnostic biomarker but was not successful in indicating the effectiveness of antibiotic treatment (205). The gut microbiome from PTLD patients was compared to healthy controls and a computer model was able to identify PTLD patients solely based upon their microbiome profile (206). While sampling and sequencing a patients’ microbiome is not a standard clinical practice, technological improvements may make this more feasible in the future. The chemokine CCL19 has potential use as a predictor for PTLD as it is elevated in patients treated for LD and then develop PTLD symptoms (90). Monitoring various host metabolites is also a prospective marker for monitoring disease progression in early LD and in identifying PTLD (207, 208). However, it is unclear if these metabolic markers could also be used to track treatment responses in these groups of patients. The use of “omics” and biomarkers to identify disease and progress is a fast-growing field of research that may hold the key for treating numerous diseases and conditions including LD and PTLD.

## 4. Conclusions/future directions

Studying PTLD in humans is severely limited due to the current inability to sufficiently identify persistent infection. It is likely that more than one mechanism may be involved in antibiotic treatment failure that leads to PTLD. These various combinations of known and unknown mechanisms and conditions may help to explain the heterogeneity and complexity of the signs and symptoms associated with PTLD. For example, spirochetes that survive after antibiotic treatment could contribute to autoimmunity or other immune-mediated responses to whole intact bacterial cells and/or their components. A constant battle between *Borrelia* and the immune system could help explain the cycle of remitting symptoms for some PTLD patients. The current treatment guidelines for LD are dependent upon the immune system’s ability to clear any persistent spirochetes, and conditional to the surviving spirochetes being non-viable and not enough to sustain a prolonged immune response. Insights and discoveries from *in vitro* experiments and animal models can translate into better patient care. It is hopeful that as personalized medicine evolves and progresses with scientific and technological discoveries and innovations, it will bring with it a true clinical cure for all LD cases thus making PTLD a thing of the past.

## Author contributions

ME and HA were responsible for the conceptualization of the manuscript. HA wrote the majority of the manuscript. ME reviewed and edited the work. All authors contributed to the article and approved the submitted version.

## Funding

Funding was provided by the Bay Area Lyme Foundation and TNPRC base grant (NIH) 5 P51 OD 011104-56.

## Conflict of interest

The authors declare that the research was conducted in the absence of any commercial or financial relationships that could be construed as a potential conflict of interest.

## Publisher’s note

All claims expressed in this article are solely those of the authors and do not necessarily represent those of their affiliated organizations, or those of the publisher, the editors and the reviewers. Any product that may be evaluated in this article, or claim that may be made by its manufacturer, is not guaranteed or endorsed by the publisher.

## References

[1] Kugeler KJ Schwartz AM Delorey MJ Mead PS Hinckley AF. Estimating the frequency of Lyme disease diagnoses, United States, 2010-2018. Emerg Infect Dis. (2021) 27:616–9. doi: 10.3201/eid2702.202731 33496229 PMC7853543

[2] Nelson CA Saha S Kugeler KJ Delorey MJ Shankar MB Hinckley AF. Incidence of clinician-diagnosed Lyme disease, United States, 2005-2010. Emerg Infect Dis. (2015) 21:1625–31. doi: 10.3201/eid2109.150417 26291194 PMC4550147

[3] Trevisan G Cinco M Trevisini S di Meo N Chersi K Ruscio M. Borreliae part 1: Borrelia Lyme group and Echidna-reptile group. Biology (Basel). (2021) 10:1036. doi: 10.3390/biology10101036 34681134 PMC8533607

[4] Strnad M Hönig V Růžek D Grubhoffer L Rego ROM. Europe-wide Meta-analysis of *Borrelia burgdorferi* Sensu Lato prevalence in questing *Ixodes ricinus* ticks. Appl Environ Microbiol. (2017) 83:e00609-17. doi: 10.1128/AEM.00609-17 28550059 PMC5514677

[5] Kurtenbach K Hanincová K Tsao JI Margos G Fish D Ogden NH. Fundamental processes in the evolutionary ecology of Lyme borreliosis. Nat Rev Microbiol. (2006) 4:660–9. doi: 10.1038/nrmicro1475 16894341

[6] Bamm VV Ko JT Mainprize IL Sanderson VP Wills MKB. Lyme disease frontiers: reconciling Borrelia biology and clinical conundrums. Pathogens. (2019) 8:299. doi: 10.3390/pathogens8040299 31888245 PMC6963551

[7] Sanchez JL. Clinical manifestations and treatment of Lyme disease. Clin Lab Med. (2015) 35:765–78. doi: 10.1016/j.cll.2015.08.004 26593256

[8] Aucott J Morrison C Munoz B Rowe PC Schwarzwalder A West SK. Diagnostic challenges of early Lyme disease: lessons from a community case series. BMC Infect Dis. (2009) 9:79. doi: 10.1186/1471-2334-9-79 19486523 PMC2698836

[9] Hirsch AG Herman RJ Rebman A Moon KA Aucott J Heaney C. Obstacles to diagnosis and treatment of Lyme disease in the USA: a qualitative study. BMJ Open. (2018) 8:e021367. doi: 10.1136/bmjopen-2017-021367 PMC6009554 29895655

[10] Moore A Nelson C Molins C Mead P Schriefer M. Current guidelines, common clinical pitfalls, and future directions for laboratory diagnosis of Lyme disease, United States. Emerg Infect Dis. (2016) 22:1169–77. doi: 10.3201/eid2207.151694 27314832 PMC4918152

[11] Lantos PM Rumbaugh J Bockenstedt LK Falck-Ytter YT Aguero-Rosenfeld ME Auwaerter PG. Clinical practice guidelines by the Infectious Diseases Society of America (IDSA), American Academy of Neurology (AAN), and American College of Rheumatology (ACR): 2020 guidelines for the prevention, diagnosis and treatment of Lyme disease. Clin Infect Dis. (2021) 72:1–8. doi: 10.1093/cid/ciab049 33483734

[12] Wormser GP Dattwyler RJ Shapiro ED Halperin JJ Steere AC Klempner MS. The clinical assessment, treatment, and prevention of Lyme disease, human granulocytic anaplasmosis, and babesiosis: clinical practice guidelines by the Infectious Diseases Society of America. Clin Infect Dis. (2006) 43:1089–134. doi: 10.1086/508667 17029130

[13] Schmidli J Hunziker T Moesli P Schaad UB. Cultivation of *Borrelia burgdorferi* from joint fluid three months after treatment of facial palsy due to Lyme borreliosis. J Infect Dis. (1988) 158:905–6. doi: 10.1093/infdis/158.4.905 3171237

[14] Cimmino MA Azzolini A Tobia F Pesce CM. Spirochetes in the spleen of a patient with chronic Lyme disease. Am J Clin Pathol. (1989) 91:95–7. doi: 10.1093/ajcp/91.1.95 2910019

[15] Bayer ME Zhang L Bayer MH. *Borrelia burgdorferi* DNA in the urine of treated patients with chronic Lyme disease symptoms. a PCR study of 97 cases. Infection. (1996) 24:347–53. doi: 10.1007/BF01716077 8923044

[16] Weber K. Treatment failure in erythema migrans--a review. Infection. (1996) 24:73–5. doi: 10.1007/BF01780663 8852475

[17] Cameron DJ. Consequences of treatment delay in Lyme disease. J Eval Clin Pract. (2007) 13:470–2. doi: 10.1111/j.1365-2753.2006.00734.x 17518818

[18] Shadick NA Phillips CB Logigian EL Steere AC Kaplan RF Berardi VP. The long-term clinical outcomes of Lyme disease. A population-based retrospective cohort study. Ann Intern Med. (1994) 121:560–7. doi: 10.7326/0003-4819-121-8-199410150-00002 8085687

[19] Asch ES Bujak DI Weiss M Peterson MG Weinstein A. Lyme disease: an infectious and postinfectious syndrome. J Rheumatol. (1994) 21:454–61. 8006888

[20] Shadick NA Phillips CB Sangha O Logigian EL Kaplan RF Wright EA. Musculoskeletal and neurologic outcomes in patients with previously treated Lyme disease. Ann Intern Med. (1999) 131:919–26. doi: 10.7326/0003-4819-131-12-199912210-00003 10610642

[21] Hirsch AG Poulsen MN Nordberg C Moon KA Rebman AW Aucott JN. Risk factors and outcomes of treatment delays in Lyme disease: a population-based retrospective cohort study. Front Med (Lausanne). (2020) 7:560018. doi: 10.3389/fmed.2020.560018 33324657 PMC7726265

[22] Aucott JN Rebman AW Crowder LA Kortte KB. Post-treatment Lyme disease syndrome symptomatology and the impact on life functioning: is there something here? Qual Life Res. (2013) 22:75–84. doi: 10.1007/s11136-012-0126-6 22294245 PMC3548099

[23] Marques A. Chronic Lyme disease: a review. Infect Dis Clin N Am. (2008) 22:341–360, vii-viii. doi: 10.1016/j.idc.2007.12.011 PMC2430045 18452806

[24] DeLong A Hsu M Kotsoris H. Estimation of cumulative number of post-treatment Lyme disease cases in the US, 2016 and 2020. BMC Public Health. (2019) 19:352. doi: 10.1186/s12889-019-6681-9 31014314 PMC6480773

[25] Cardenas-de la Garza JA De la Cruz-Valadez E Ocampo-Candiani J Welsh O. Clinical spectrum of Lyme disease. Eur J Clin Microbiol Infect Dis. (2019) 38:201–8. doi: 10.1007/s10096-018-3417-1 30456435

[26] Shor S Green C Szantyr B Phillips S Liegner K Burrascano JJ. Chronic Lyme disease: an evidence-based definition by the ILADS working group. Antibiotics (Basel). (2019) 8:269. doi: 10.3390/antibiotics8040269 31888310 PMC6963229

[27] Horowitz RI Freeman PR. Precision medicine: the role of the MSIDS model in defining, diagnosing, and treating chronic Lyme disease/post treatment Lyme disease syndrome and other chronic illness: part 2. Healthcare (Basel). (2018) 6:129. doi: 10.3390/healthcare6040129 30400667 PMC6316761

[28] Rebman AW Yang T Aucott JN. Symptom heterogeneity and patient subgroup classification among US patients with post-treatment Lyme disease: an observational study. BMJ Open. (2021) 11:e040399. doi: 10.1136/bmjopen-2020-040399 PMC7812114 33441353

[29] Auwaerter PG. Point: antibiotic therapy is not the answer for patients with persisting symptoms attributable to Lyme disease. Clin Infect Dis. (2007) 45:143–8. doi: 10.1086/518854 17578771

[30] Stricker RB. Counterpoint: long-term antibiotic therapy improves persistent symptoms associated with Lyme disease. Clin Infect Dis. (2007) 45:149–57. doi: 10.1086/518853 17578772

[31] Kiriakidou M Ching CL. Systemic lupus erythematosus. Ann Intern Med. (2020) 172:ITC81–96. doi: 10.7326/AITC202006020 32479157

[32] Fichtner ML Jiang R Bourke A Nowak RJ O’Connor KC. Autoimmune pathology in myasthenia gravis disease subtypes is governed by divergent mechanisms of immunopathology. Front Immunol. (2020) 11:776. doi: 10.3389/fimmu.2020.00776 32547535 PMC7274207

[33] Antonelli A Fallahi P Elia G Ragusa F Paparo SR Ruffilli I. Graves’ disease: clinical manifestations, immune pathogenesis (cytokines and chemokines) and therapy. Best Pract Res Clin Endocrinol Metab. (2020) 34:101388. doi: 10.1016/j.beem.2020.101388 32059832

[34] Larabi A Barnich N Nguyen HTT. New insights into the interplay between autophagy, gut microbiota and inflammatory responses in IBD. Autophagy. (2020) 16:38–51. doi: 10.1080/15548627.2019.1635384 31286804 PMC6984609

[35] Shahrizaila N Lehmann HC Kuwabara S. Guillain-Barré syndrome. Lancet. (2021) 397:1214–28. doi: 10.1016/S0140-6736(21)00517-1 33647239

[36] Murúa SR Farez MF Quintana FJ. The immune response in multiple sclerosis. Annu Rev Pathol. (2022) 17:121–39. doi: 10.1146/annurev-pathol-052920-040318 34606377

[37] Zhao H Zhang W Cheng D You L Huang Y Lu Y. Investigating dysbiosis and microbial treatment strategies in inflammatory bowel disease based on two modified Koch’s postulates. Front Med (Lausanne). (2022) 9:1023896. doi: 10.3389/fmed.2022.1023896 36438062 PMC9684636

[38] Theofilopoulos AN Kono DH Baccala R. The multiple pathways to autoimmunity. Nat Immunol. (2017) 18:716–24. doi: 10.1038/ni.3731 28632714 PMC5791156

[39] Blackburn KM Wang C. Post-infectious neurological disorders. Ther Adv Neurol Disord. (2020) 13:1756286420952901. doi: 10.1177/1756286420952901 32944082 PMC7466892

[40] Collins C Peltz G. Immunoreactive epitopes on an expressed recombinant flagellar protein of *Borrelia burgdorferi*. Infect Immun. (1991) 59:514–20. doi: 10.1128/iai.59.2.514-520.1991 1702766 PMC257779

[41] Ehrenstein M Isenberg D. Autoimmunity associated with infection: leprosy, acute rheumatic fever and Lyme disease. Curr Opin Immunol. (1991) 3:930–5. doi: 10.1016/s0952-7915(05)80016-0 1793538

[42] Mensi N Webb DR Turck CW Peltz GA. Characterization of *Borrelia burgdorferi* proteins reactive with antibodies in synovial fluid of a patient with Lyme arthritis. Infect Immun. (1990) 58:2404–7. doi: 10.1128/iai.58.7.2404-2407.1990 2365463 PMC258829

[43] Steere AC Dwyer E Winchester R. Association of chronic Lyme arthritis with HLA-DR4 and HLA-DR2 alleles. N Engl J Med. (1990) 323:219–23. doi: 10.1056/NEJM199007263230402 2078208

[44] Steere AC Klitz W Drouin EE Falk BA Kwok WW Nepom GT. Antibiotic-refractory Lyme arthritis is associated with HLA-DR molecules that bind a *Borrelia burgdorferi* peptide. J Exp Med. (2006) 203:961–71. doi: 10.1084/jem.20052471 16585267 PMC3212725

[45] Kalish RA Leong JM Steere AC. Association of treatment-resistant chronic Lyme arthritis with HLA-DR4 and antibody reactivity to OspA and OspB of *Borrelia burgdorferi*. Infect Immun. (1993) 61:2774–9. 7685738 10.1128/iai.61.7.2774-2779.1993 PMC280920

[46] Gross DM Forsthuber T Tary-Lehmann M Etling C Ito K Nagy ZA. Identification of LFA-1 as a candidate autoantigen in treatment-resistant Lyme arthritis. Science. (1998) 281:703–6. doi: 10.1126/science.281.5377.703 9685265

[47] Trollmo C Meyer AL Steere AC Hafler DA Huber BT. Molecular mimicry in Lyme arthritis demonstrated at the single cell level: LFA-1 alpha L is a partial agonist for outer surface protein A-reactive T cells. J Immunol. (2001) 166:5286–91. doi: 10.4049/jimmunol.166.8.5286 11290815

[48] Crowley JT Drouin EE Pianta A Strle K Wang Q Costello CE. A highly expressed human protein, Apolipoprotein B-100, serves as an autoantigen in a subgroup of patients with Lyme disease. J Infect Dis. (2015) 212:1841–50. doi: 10.1093/infdis/jiv310 26014802 PMC4633766

[49] Crowley JT Strle K Drouin EE Pianta A Arvikar SL Wang Q. Matrix metalloproteinase-10 is a target of T and B cell responses that correlate with synovial pathology in patients with antibiotic-refractory Lyme arthritis. J Autoimmun. (2016) 69:24–37. doi: 10.1016/j.jaut.2016.02.005 26922382 PMC4826816

[50] Drouin EE Seward RJ Strle K McHugh G Katchar K Londoño D. A novel human autoantigen, endothelial cell growth factor, is a target of T and B cell responses in patients with Lyme disease. Arthritis Rheum. (2013) 65:186–96. doi: 10.1002/art.37732 23044924 PMC3535550

[51] Pianta A Drouin EE Crowley JT Arvikar S Strle K Costello CE. Annexin A2 is a target of autoimmune T and B cell responses associated with synovial fibroblast proliferation in patients with antibiotic-refractory Lyme arthritis. Clin Immunol. (2015) 160:336–41. doi: 10.1016/j.clim.2015.07.005 26187145 PMC4582008

[52] Maier B Molinger M Cope AP Fugger L Schneider-Mergener J Sønderstrup G. Multiple cross-reactive self-ligands for *Borrelia burgdorferi*-specific HLA-DR4-restricted T cells. Eur J Immunol. (2000) 30:448–57. doi: 10.1002/1521-4141(200002)30:2<448::AID-IMMU448>3.0.CO;2-9 10671200

[53] Whiteside SK Snook JP Ma Y Sonderegger FL Fisher C Petersen C. IL-10 deficiency reveals a role for TLR2-dependent bystander activation of T cells in Lyme arthritis. J Immunol. (2018) 200:1457–70. doi: 10.4049/jimmunol.1701248 29330323 PMC5809275

[54] Shen S Shin JJ Strle K McHugh G Li X Glickstein LJ. Treg cell numbers and function in patients with antibiotic-refractory or antibiotic-responsive Lyme arthritis. Arthritis Rheum. (2010) 62:2127–37. doi: 10.1002/art.27468 20506317 PMC2913315

[55] Siebers EM Liedhegner ES Lawlor MW Schell RF Nardelli DT. Regulatory T cells contribute to resistance against Lyme arthritis. Infect Immun. (2020) 88:e00160-20. doi: 10.1128/IAI.00160-20 32778610 PMC7573436

[56] Strle K Sulka KB Pianta A Crowley JT Arvikar SL Anselmo A. T-helper 17 cell cytokine responses in Lyme disease correlate with *Borrelia burgdorferi* antibodies during early infection and with autoantibodies late in the illness in patients with antibiotic-refractory Lyme arthritis. Clin Infect Dis. (2017) 64:930–8. doi: 10.1093/cid/cix002 28077518 PMC5850331

[57] Elliott SE Kongpachith S Lingampalli N Adamska JZ Cannon BJ Mao R. Affinity maturation drives epitope spreading and generation of Proinflammatory anti-Citrullinated protein antibodies in rheumatoid arthritis. Arthritis Rheumatol. (2018) 70:1946–58. doi: 10.1002/art.40587 29927104 PMC6261684

[58] Steere AC. Treatment of Lyme arthritis. J Rheumatol. (2019) 46:871–3. doi: 10.3899/jrheum.190320 31371661

[59] Hemmer B Gran B Zhao Y Marques A Pascal J Tzou A. Identification of candidate T-cell epitopes and molecular mimics in chronic Lyme disease. Nat Med. (1999) 5:1375–82. doi: 10.1038/70946 10581079

[60] Martin R Gran B Zhao Y Markovic-Plese S Bielekova B Marques A. Molecular mimicry and antigen-specific T cell responses in multiple sclerosis and chronic CNS Lyme disease. J Autoimmun. (2001) 16:187–92. doi: 10.1006/jaut.2000.0501 11334482

[61] Raveche ES Schutzer SE Fernandes H Bateman H McCarthy BA Nickell SP. Evidence of Borrelia autoimmunity-induced component of Lyme carditis and arthritis. J Clin Microbiol. (2005) 43:850–6. doi: 10.1128/jcm.43.2.850-856.2005 15695691 PMC548028

[62] Sulka KB Strle K Crowley JT Lochhead RB Anthony R Steere AC. Correlation of Lyme disease-associated IgG4 autoantibodies with synovial pathology in antibiotic-refractory Lyme arthritis. Arthritis Rheumatol. (2018) 70:1835–46. doi: 10.1002/art.40566 29790305 PMC6203610

[63] Strle K Stupica D Drouin EE Steere AC Strle F. Elevated levels of IL-23 in a subset of patients with post-Lyme disease symptoms following erythema migrans. Clin Infect Dis. (2014) 58:372–80. doi: 10.1093/cid/cit735 24218102 PMC3890340

[64] Chandra A Wormser GP Klempner MS Trevino RP Crow MK Latov N. Anti-neural antibody reactivity in patients with a history of Lyme borreliosis and persistent symptoms. Brain Behav Immun. (2010) 24:1018–24. doi: 10.1016/j.bbi.2010.03.002 20227484 PMC2897967

[65] Chandra A Wormser GP Marques AR Latov N Alaedini A. Anti-*Borrelia burgdorferi* antibody profile in post-Lyme disease syndrome. Clin Vaccine Immunol. (2011) 18:767–71. doi: 10.1128/cvi.00002-11 21411605 PMC3122515

[66] Jacek E Fallon BA Chandra A Crow MK Wormser GP Alaedini A. Increased IFNα activity and differential antibody response in patients with a history of Lyme disease and persistent cognitive deficits. J Neuroimmunol. (2013) 255:85–91. doi: 10.1016/j.jneuroim.2012.10.011 23141748 PMC3557545

[67] Maccallini P Bonin S Trevisan G. Autoimmunity against a glycolytic enzyme as a possible cause for persistent symptoms in Lyme disease. Med Hypotheses. (2018) 110:1–8. doi: 10.1016/j.mehy.2017.10.024 29317049

[68] Klempner MS Wormser GH Wade K Trevino RP Tang J Kaslow RA. A case-control study to examine HLA haplotype associations in patients with posttreatment chronic Lyme disease. J Infect Dis. (2005) 192:1010–3. doi: 10.1086/432733 16107953

[69] Coburn J Garcia B Hu LT Jewett MW Kraiczy P Norris SJ. Lyme disease pathogenesis. Curr Issues Mol Biol. (2021) 42:473–518. doi: 10.21775/cimb.042.473 33353871 PMC8046170

[70] Bockenstedt LK Wooten RM Baumgarth N. Immune response to *Borrelia*: lessons from Lyme disease spirochetes. Curr Issues Mol Biol. (2021) 42:145–90. doi: 10.21775/cimb.042.145 33289684 PMC10842262

[71] Weitzner E McKenna D Nowakowski J Scavarda C Dornbush R Bittker S. Long-term assessment of post-treatment symptoms in patients with culture-confirmed early Lyme disease. Clin Infect Dis. (2015) 61:1800–6. doi: 10.1093/cid/civ735 26385994 PMC4657537

[72] Bouquet J Soloski MJ Swei A Cheadle C Federman S Billaud JN. Longitudinal Transcriptome analysis reveals a sustained differential gene expression signature in patients treated for acute Lyme disease. MBio. (2016) 7:e00100-16. doi: 10.1128/mBio.00100-16 26873097 PMC4791844

[73] Barriales D Martín-Ruiz I Carreras-González A Montesinos-Robledo M Azkargorta M Iloro I. *Borrelia burgdorferi* infection induces long-term memory-like responses in macrophages with tissue-wide consequences in the heart. PLoS Biol. (2021) 19:e3001062. doi: 10.1371/journal.pbio.3001062 33395408 PMC7808612

[74] Ramesh G Borda JT Dufour J Kaushal D Ramamoorthy R Lackner AA. Interaction of the Lyme disease spirochete *Borrelia burgdorferi* with brain parenchyma elicits inflammatory mediators from glial cells as well as glial and neuronal apoptosis. Am J Pathol. (2008) 173:1415–27. doi: 10.2353/ajpath.2008.080483 18832582 PMC2570132

[75] Ramesh G Borda JT Gill A Ribka EP Morici LA Mottram P. Possible role of glial cells in the onset and progression of Lyme neuroborreliosis. J Neuroinflammation. (2009) 6:23. doi: 10.1186/1742-2094-6-23 19706181 PMC2748066

[76] Ramesh G Didier PJ England JD Santana-Gould L Doyle-Meyers LA Martin DS. Inflammation in the pathogenesis of Lyme neuroborreliosis. Am J Pathol. (2015) 185:1344–60. doi: 10.1016/j.ajpath.2015.01.024 25892509 PMC4419210

[77] Hoffman PN Lasek RJ. Axonal transport of the cytoskeleton in regenerating motor neurons: constancy and change. Brain Res. (1980) 202:317–33. doi: 10.1016/0006-8993(80)90144-4 6159947

[78] Radolf JD Strle K Lemieux JE Strle F. Lyme disease in humans. Curr Issues Mol Biol. (2021) 42:333–84. doi: 10.21775/cimb.042.333 33303701 PMC7946767

[79] Miller JC Ma Y Bian J Sheehan KC Zachary JF Weis JH. A critical role for type I IFN in arthritis development following *Borrelia burgdorferi* infection of mice. J Immunol. (2008) 181:8492–503. doi: 10.4049/jimmunol.181.12.8492 19050267 PMC3024833

[80] Miller JC Ma Y Crandall H Wang X Weis JJ. Gene expression profiling provides insights into the pathways involved in inflammatory arthritis development: murine model of Lyme disease. Exp Mol Pathol. (2008) 85:20–7. doi: 10.1016/j.yexmp.2008.03.004 18462718 PMC2565650

[81] Wang G Ma Y Buyuk A McClain S Weis JJ Schwartz I. Impaired host defense to infection and toll-like receptor 2-independent killing of *Borrelia burgdorferi* clinical isolates in TLR2-deficient C3H/HeJ mice. FEMS Microbiol Lett. (2004) 231:219–25. doi: 10.1016/S0378-1097(03)00960-1 14987768

[82] Casselli T Divan A Vomhof-DeKrey EE Tourand Y Pecoraro HL Brissette CA. A murine model of Lyme disease demonstrates that *Borrelia burgdorferi* colonizes the dura mater and induces inflammation in the central nervous system. PLoS Pathog. (2021) 17:e1009256. doi: 10.1371/journal.ppat.1009256 33524035 PMC7877756

[83] Ma Y Seiler KP Tai KF Yang L Woods M Weis JJ. Outer surface lipoproteins of *Borrelia burgdorferi* stimulate nitric oxide production by the cytokine-inducible pathway. Infect Immun. (1994) 62:3663–71. doi: 10.1128/iai.62.9.3663-3671.1994 7520417 PMC303016

[84] Sjöwall J Fryland L Nordberg M Sjögren F Garpmo U Jansson C. Decreased Th1-type inflammatory cytokine expression in the skin is associated with persisting symptoms after treatment of erythema migrans. PLoS One. (2011) 6:e18220. doi: 10.1371/journal.pone.0018220 21483819 PMC3069060

[85] Lochhead RB Arvikar SL Aversa JM Sadreyev RI Strle K Steere AC. Robust interferon signature and suppressed tissue repair gene expression in synovial tissue from patients with postinfectious, *Borrelia burgdorferi*-induced Lyme arthritis. Cell Microbiol. (2019) 21:e12954. doi: 10.1111/cmi.12954 30218476 PMC6724218

[86] Lochhead RB Ordoñez D Arvikar SL Aversa JM Oh LS Heyworth B. Interferon-gamma production in Lyme arthritis synovial tissue promotes differentiation of fibroblast-like synoviocytes into immune effector cells. Cell Microbiol. (2019) 21:e12992. doi: 10.1111/cmi.12992 30550623 PMC6336510

[87] Lochhead RB Zachary JF Dalla Rosa L Ma Y Weis JH O’Connell RM. Antagonistic interplay between MicroRNA-155 and IL-10 during Lyme Carditis and arthritis. PLoS One. (2015) 10:e0135142. doi: 10.1371/journal.pone.0135142 26252010 PMC4529177

[88] Lochhead RB Ma Y Zachary JF Baltimore D Zhao JL Weis JH. MicroRNA-146a provides feedback regulation of Lyme arthritis but not carditis during infection with *Borrelia burgdorferi*. PLoS Pathog. (2014) 10:e1004212. doi: 10.1371/journal.ppat.1004212 24967703 PMC4072785

[89] Brown CR Blaho VA Fritsche KL Loiacono CM. Stat1 deficiency exacerbates carditis but not arthritis during experimental Lyme borreliosis. J Interf Cytokine Res. (2006) 26:390–9. doi: 10.1089/jir.2006.26.390 16734559

[90] Aucott JN Soloski MJ Rebman AW Crowder LA Lahey LJ Wagner CA. CCL19 as a chemokine risk factor for Posttreatment Lyme disease syndrome: a prospective clinical cohort study. Clin Vaccine Immunol. (2016) 23:757–66. doi: 10.1128/CVI.00071-16 27358211 PMC5014924

[91] Sahay B Bashant K Nelson NLJ Patsey RL Gadila SK Boohaker R. Induction of interleukin 10 by *Borrelia burgdorferi* is regulated by the action of CD14-dependent p38 mitogen-activated protein kinase and cAMP-mediated chromatin remodeling. Infect Immun. (2018) 86:e00781-17. doi: 10.1128/IAI.00781-17 29311239 PMC5865024

[92] Barthold SW Sidman CL Smith AL. Lyme borreliosis in genetically resistant and susceptible mice with severe combined immunodeficiency. Am J Trop Med Hyg. (1992) 47:605–13. doi: 10.4269/ajtmh.1992.47.605 1449201

[93] Schaible UE Gay S Museteanu C Kramer MD Zimmer G Eichmann K. Lyme borreliosis in the severe combined immunodeficiency (SCID) mouse manifests predominantly in the joints, heart, and liver. Am J Pathol. (1990) 137:811–20. PMID: 2221014 PMC1877559

[94] Wang X Ma Y Weis JH Zachary JF Kirschning CJ Weis JJ. Relative contributions of innate and acquired host responses to bacterial control and arthritis development in Lyme disease. Infect Immun. (2005) 73:657–60. doi: 10.1128/IAI.73.1.657-660.2005 15618212 PMC538980

[95] Blum LK Adamska JZ Martin DS Rebman AW Elliott SE Cao RRL. Robust B cell responses predict rapid resolution of Lyme disease. Front Immunol. (2018) 9:1634. doi: 10.3389/fimmu.2018.01634 30072990 PMC6060717

[96] Elsner RA Hastey CJ Baumgarth N. CD4+ T cells promote antibody production but not sustained affinity maturation during *Borrelia burgdorferi* infection. Infect Immun. (2015) 83:48–56. doi: 10.1128/iai.02471-14 25312948 PMC4288900

[97] Meriläinen L Brander H Herranen A Schwarzbach A Gilbert L. Pleomorphic forms of *Borrelia burgdorferi* induce distinct immune responses. Microbes Infect. (2016) 18:484–95. doi: 10.1016/j.micinf.2016.04.002 27139815

[98] Feng J Li T Yee R Yuan Y Bai C Cai M. Stationary phase persister/biofilm microcolony of *Borrelia burgdorferi* causes more severe disease in a mouse model of Lyme arthritis: implications for understanding persistence, post-treatment Lyme disease syndrome (PTLDS), and treatment failure. Discov Med. (2019) 27:125–38. 30946803

[99] Yrjänäinen H Hytönen J Hartiala P Oksi J Viljanen MK. Persistence of borrelial DNA in the joints of *Borrelia burgdorferi*-infected mice after ceftriaxone treatment. APMIS. (2010) 118:665–73. doi: 10.1111/j.1600-0463.2010.02615.x 20718718

[100] Iyer R Mukherjee P Wang K Simons J Wormser GP Schwartz I. Detection of *Borrelia burgdorferi* nucleic acids after antibiotic treatment does not confirm viability. J Clin Microbiol. (2013) 51:857–62. doi: 10.1128/JCM.02785-12 23269733 PMC3592055

[101] Salo J Jaatinen A Söderström M Viljanen MK Hytönen J. Decorin binding proteins of *Borrelia burgdorferi* promote arthritis development and joint specific post-treatment DNA persistence in mice. PLoS One. (2015) 10:e0121512. doi: 10.1371/journal.pone.0121512 25816291 PMC4376631

[102] Jutras BL Lochhead RB Kloos ZA Biboy J Strle K Booth CJ. Peptidoglycan is a persistent antigen in patients with Lyme arthritis. Proc Natl Acad Sci U S A. (2019) 116:13498–507. doi: 10.1073/pnas.1904170116 31209025 PMC6613144

[103] Embers ME Ramamoorthy R Philipp MT. Survival strategies of *Borrelia burgdorferi*, the etiologic agent of Lyme disease. Microbes Infect. (2004) 6:312–8. doi: 10.1016/j.micinf.2003.11.014 15065567

[104] Barthold SW Persing DH Armstrong AL Peeples RA. Kinetics of *Borrelia burgdorferi* dissemination and evolution of disease after intradermal inoculation of mice. Am J Pathol. (1991) 139:263–73. 1867318 PMC1886084

[105] Chang YF Ku YW Chang CF Chang CD McDonough SP Divers T. Antibiotic treatment of experimentally *Borrelia burgdorferi*-infected ponies. Vet Microbiol. (2005) 107:285–94. doi: 10.1016/j.vetmic.2005.02.006 15863289

[106] Straubinger RK Summers BA Chang YF Appel MJ. Persistence of *Borrelia burgdorferi* in experimentally infected dogs after antibiotic treatment. J Clin Microbiol. (1997) 35:111–6. 8968890 10.1128/jcm.35.1.111-116.1997 PMC229521

[107] Embers ME Hasenkampf NR Jacobs MB Philipp MT. Dynamic longitudinal antibody responses during *Borrelia burgdorferi* infection and antibiotic treatment of rhesus macaques. Clin Vaccine Immunol. (2012) 19:1218–26. doi: 10.1128/CVI.00228-12 22718128 PMC3416093

[108] Bigger JW. Treatment of staphylococcal infections with penicillin by intermittent sterilisation. Lancet. (1944) 244:497–500. doi: 10.1016/S0140-6736(00)74210-3

[109] Fisher RA Gollan B Helaine S. Persistent bacterial infections and persister cells. Nat Rev Microbiol. (2017) 15:453–64. doi: 10.1038/nrmicro.2017.42 28529326

[110] Balaban NQ Merrin J Chait R Kowalik L Leibler S. Bacterial persistence as a phenotypic switch. Science. (2004) 305:1622–5. doi: 10.1126/science.1099390 15308767

[111] Kussell E Kishony R Balaban NQ Leibler S. Bacterial persistence: a model of survival in changing environments. Genetics. (2005) 169:1807–14. doi: 10.1534/genetics.104.035352 15687275 PMC1449587

[112] Balaban NQ Helaine S Lewis K Ackermann M Aldridge B Andersson DI. Definitions and guidelines for research on antibiotic persistence. Nat Rev Microbiol. (2019) 17:441–8. doi: 10.1038/s41579-019-0196-3 30980069 PMC7136161

[113] Liegner KB Shapiro JR Ramsay D Halperin AJ Hogrefe W Kong L. Recurrent erythema migrans despite extended antibiotic treatment with minocycline in a patient with persisting *Borrelia burgdorferi* infection. J Am Acad Dermatol. (1993) 28:312–4. doi: 10.1016/0190-9622(93)70043-s 8436647

[114] Preac-Mursic V Weber K Pfister HW Wilske B Gross B Baumann A. Survival of *Borrelia burgdorferi* in antibiotically treated patients with Lyme borreliosis. Infection. (1989) 17:355–9. doi: 10.1007/bf01645543 2613324

[115] Sharma B Brown AV Matluck NE Hu LT Lewis K. *Borrelia burgdorferi*, the causative agent of Lyme disease, forms drug-tolerant Persister cells. Antimicrob Agents Chemother. (2015) 59:4616–24. doi: 10.1128/AAC.00864-15 26014929 PMC4505243

[116] Caskey JR Embers ME. Persister development by *Borrelia burgdorferi* populations in vitro. Antimicrob Agents Chemother. (2015) 59:6288–95. doi: 10.1128/AAC.00883-15 26248368 PMC4576077

[117] Feng J Auwaerter PG Zhang Y. Drug combinations against *Borrelia burgdorferi* persisters in vitro: eradication achieved by using daptomycin, cefoperazone and doxycycline. PLoS One. (2015) 10:e0117207. doi: 10.1371/journal.pone.0117207 25806811 PMC4373819

[118] Kaldalu N Tenson T. Slow growth causes bacterial persistence. Sci Signal. (2019) 12:eaay1167. doi: 10.1126/scisignal.aay1167 31363066

[119] Straubinger RK Straubinger AF Summers BA Jacobson RH. Status of *Borrelia burgdorferi* infection after antibiotic treatment and the effects of corticosteroids: an experimental study. J Infect Dis. (2000) 181:1069–81. doi: 10.1086/315340 10720533

[120] Straubinger RK. PCR-based quantification of *Borrelia burgdorferi* organisms in canine tissues over a 500-day postinfection period. J Clin Microbiol. (2000) 38:2191–9. doi: 10.1128/JCM.38.6.2191-2199.2000 10834975 PMC86761

[121] Barthold SW Hodzic E Imai DM Feng S Yang X Luft BJ. Ineffectiveness of tigecycline against persistent *Borrelia burgdorferi*. Antimicrob Agents Chemother. (2010) 54:643–51. doi: 10.1128/AAC.00788-09 19995919 PMC2812145

[122] Bockenstedt LK Mao J Hodzic E Barthold SW Fish D. Detection of attenuated, noninfectious spirochetes in *Borrelia burgdorferi*-infected mice after antibiotic treatment. J Infect Dis. (2002) 186:1430–7. doi: 10.1086/345284 12404158

[123] Hodzic E Feng S Holden K Freet KJ Barthold SW. Persistence of *Borrelia burgdorferi* following antibiotic treatment in mice. Antimicrob Agents Chemother. (2008) 52:1728–36. doi: 10.1128/AAC.01050-07 18316520 PMC2346637

[124] Hodzic E Imai D Feng S Barthold SW. Resurgence of persisting non-cultivable *Borrelia burgdorferi* following antibiotic treatment in mice. PLoS One. (2014) 9:e86907. doi: 10.1371/journal.pone.0086907 24466286 PMC3900665

[125] Hodzic E Imai DM Escobar E. Generality of post-antimicrobial treatment persistence of *Borrelia burgdorferi* strains N40 and B31 in genetically susceptible and resistant mouse strains. Infect Immun. (2019) 87:e00442-19. doi: 10.1128/IAI.00442-19 31308087 PMC6759297

[126] Embers ME Hasenkampf NR Jacobs MB Tardo AC Doyle-Meyers LA Philipp MT. Variable manifestations, diverse seroreactivity and post-treatment persistence in non-human primates exposed to *Borrelia burgdorferi* by tick feeding. PLoS One. (2017) 12:e0189071. doi: 10.1371/journal.pone.0189071 29236732 PMC5728523

[127] Crossland NA Alvarez X Embers ME. Late disseminated Lyme disease: associated pathology and spirochete persistence Posttreatment in Rhesus macaques. Am J Pathol. (2018) 188:672–82. doi: 10.1016/j.ajpath.2017.11.005 29242055 PMC5840488

[128] Marques A Telford SR Turk SP Chung E Williams C Dardick K. Xenodiagnosis to detect *Borrelia burgdorferi* infection: a first-in-human study. Clin Infect Dis. (2014) 58:937–45. doi: 10.1093/cid/cit939 24523212 PMC3952603

[129] Pavia CS Wormser GP. Culture of the entire mouse to determine whether cultivable *Borrelia burgdorferi* persists in infected mice treated with a five-day course of ceftriaxone. Antimicrob Agents Chemother. (2014) 58:6701–3. doi: 10.1128/AAC.03751-14 25155590 PMC4249442

[130] Sharma B McCarthy JE Freliech CA Clark MM Hu LT. Genetic background amplifies the effect of immunodeficiency in antibiotic efficacy against *Borrelia burgdorferi*. J Infect Dis. (2021) 224:345–50. doi: 10.1093/infdis/jiaa719 33216133 PMC8280483

[131] Bockenstedt LK Gonzalez DG Haberman AM Belperron AA. Spirochete antigens persist near cartilage after murine Lyme borreliosis therapy. J Clin Invest. (2012) 122:2652–60. doi: 10.1172/JCI58813 22728937 PMC3386809

[132] Zhang Y. Persisters, persistent infections and the yin-Yang model. Emerg Microbes Infect. (2014) 3:e3. doi: 10.1038/emi.2014.3 26038493 PMC3913823

[133] Wormser GP Schwartz I. Antibiotic treatment of animals infected with *Borrelia burgdorferi*. Clin Microbiol Rev. (2009) 22:387–95. doi: 10.1128/CMR.00004-09 19597005 PMC2708393

[134] Wormser GP O’Connell S Pachner AR Schwartz I Shapiro ED Stanek G. Critical analysis of a doxycycline treatment trial of rhesus macaques infected with *Borrelia burgdorferi*. Diagn Microbiol Infect Dis. (2018) 92:183–8. doi: 10.1016/j.diagmicrobio.2018.06.007 30017315 PMC6173987

[135] Shapiro ED. Repeat or persistent Lyme disease: persistence, recrudescence or reinfection with *Borrelia Burgdorferi*? F1000Prime Rep. (2015) 7:11. doi: 10.12703/P7-11 25705394 PMC4311275

[136] Cruz AR Moore MW La Vake CJ Eggers CH Salazar JC Radolf JD. Phagocytosis of *Borrelia burgdorferi*, the Lyme disease spirochete, potentiates innate immune activation and induces apoptosis in human monocytes. Infect Immun. (2008) 76:56–70. doi: 10.1128/IAI.01039-07 17938216 PMC2223637

[137] Salazar JC Duhnam-Ems S La Vake C Cruz AR Moore MW Caimano MJ. Activation of human monocytes by live *Borrelia burgdorferi* generates TLR2-dependent and-independent responses which include induction of IFN-beta. PLoS Pathog. (2009) 5:e1000444. doi: 10.1371/journal.ppat.1000444 19461888 PMC2679197

[138] Parthasarathy G Gadila SKG. Neuropathogenicity of non-viable *Borrelia burgdorferi* ex vivo. Sci Rep. (2022) 12:688. doi: 10.1038/s41598-021-03837-0 35027599 PMC8758786

[139] Gadila SKG Rosoklija G Dwork AJ Fallon BA Embers ME. Detecting *Borrelia* spirochetes: a case study with validation among autopsy specimens. Front Neurol. (2021) 12:628045. doi: 10.3389/fneur.2021.628045 34040573 PMC8141553

[140] Caskey JR Hasenkampf NR Martin DS Chouljenko VN Subramanian R Cheslock MA. The functional and molecular effects of doxycycline treatment on *Borrelia burgdorferi* phenotype. Front Microbiol. (2019) 10:690. doi: 10.3389/fmicb.2019.00690 31057493 PMC6482230

[141] Cabello FC Embers ME Newman SA Godfrey HP. Borreliella burgdorferi antimicrobial-tolerant persistence in Lyme disease and posttreatment Lyme disease syndromes. mBio. (2022) 13:e0344021. doi: 10.1128/mbio.03440-21 35467428 PMC9239140

[142] Cabello FC Godfrey HP Bugrysheva JV Newman SA. Sleeper cells: the stringent response and persistence in the Borreliella (*Borrelia*) burgdorferi enzootic cycle. Environ Microbiol. (2017) 19:3846–62. doi: 10.1111/1462-2920.13897 28836724 PMC5794220

[143] Feng J Shi W Zhang S Zhang Y. Persister mechanisms in *Borrelia burgdorferi*: implications for improved intervention. Emerg Microbes Infect. (2015) 4:e51. doi: 10.1038/emi.2015.51 26954994 PMC5176083

[144] Kersten A Poitschek C Rauch S Aberer E. Effects of penicillin, ceftriaxone, and doxycycline on morphology of *Borrelia burgdorferi*. Antimicrob Agents Chemother. (1995) 39:1127–33. doi: 10.1128/AAC.39.5.1127 7625800 PMC162695

[145] Murgia R Cinco M. Induction of cystic forms by different stress conditions in *Borrelia burgdorferi*. APMIS. (2004) 112:57–62. doi: 10.1111/j.1600-0463.2004.apm1120110.x 14961976

[146] Sapi E Bastian SL Mpoy CM Scott S Rattelle A Pabbati N. Characterization of biofilm formation by *Borrelia burgdorferi* in vitro. PLoS One. (2012) 7:e48277. doi: 10.1371/journal.pone.0048277 23110225 PMC3480481

[147] Meriläinen L Herranen A Schwarzbach A Gilbert L. Morphological and biochemical features of *Borrelia burgdorferi* pleomorphic forms. Microbiology (Reading). (2015) 161:516–27. doi: 10.1099/mic.0.000027 25564498 PMC4339653

[148] Alban PS Johnson PW Nelson DR. Serum-starvation-induced changes in protein synthesis and morphology of *Borrelia burgdorferi*. Microbiology (Reading). (2000) 146:119–27. doi: 10.1099/00221287-146-1-119 10658658

[149] Brorson O Brorson SH. Transformation of cystic forms of *Borrelia burgdorferi* to normal, mobile spirochetes. Infection. (1997) 25:240–6. doi: 10.1007/BF01713153 9266264

[150] Miklossy J Kasas S Zurn AD McCall S Yu S McGeer PL. Persisting atypical and cystic forms of *Borrelia burgdorferi* and local inflammation in Lyme neuroborreliosis. J Neuroinflammation. (2008) 5:40. doi: 10.1186/1742-2094-5-40 18817547 PMC2564911

[151] Lantos PM Auwaerter PG Wormser GP. A systematic review of *Borrelia burgdorferi* morphologic variants does not support a role in chronic Lyme disease. Clin Infect Dis. (2014) 58:663–71. doi: 10.1093/cid/cit810 24336823 PMC3922218

[152] Srivastava SY de Silva AM. Characterization of *Borrelia burgdorferi* aggregates. Vector Borne Zoonotic Dis. (2009) 9:323–9. doi: 10.1089/vbz.2008.0148 19499997 PMC2904187

[153] Sapi E Kaur N Anyanwu S Luecke DF Datar A Patel S. Evaluation of in-vitro antibiotic susceptibility of different morphological forms of *Borrelia burgdorferi*. Infect Drug Resist. (2011) 4:97–113. doi: 10.2147/IDR.S19201 21753890 PMC3132871

[154] Berndtson K. Review of evidence for immune evasion and persistent infection in Lyme disease. Int J Gen Med. (2013) 6:291–306. doi: 10.2147/IJGM.S44114 23637552 PMC3636972

[155] Kraiczy P. Hide and Seek: how Lyme disease spirochetes overcome complement attack. Front Immunol. (2016) 7:385. doi: 10.3389/fimmu.2016.00385 27725820 PMC5036304

[156] Klempner MS Noring R Rogers RA. Invasion of human skin fibroblasts by the Lyme disease spirochete, *Borrelia burgdorferi*. J Infect Dis. (1993) 167:1074–81. doi: 10.1093/infdis/167.5.1074 8486939

[157] Karvonen K Nykky J Marjomäki V Gilbert L. Distinctive evasion mechanisms to allow persistence of. Front Microbiol. (2021) 12:711291. doi: 10.3389/fmicb.2021.711291 34712208 PMC8546339

[158] Stricker RB Lautin A Burrascano JJ. Lyme disease: point/counterpoint. Expert Rev Anti-Infect Ther. (2005) 3:155–65. doi: 10.1586/14787210.3.2.155 15918774

[159] DeLong AK Blossom B Maloney E Phillips SE. Potential benefits of retreatment highlight the need for additional Lyme disease research. Am J Med. (2014) 127:e9–e10. doi: 10.1016/j.amjmed.2013.08.028 24462019

[160] Sjöwall J Ledel A Ernerudh J Ekerfelt C Forsberg P. Doxycycline-mediated effects on persistent symptoms and systemic cytokine responses post-neuroborreliosis: a randomized, prospective, cross-over study. BMC Infect Dis. (2012) 12:186. doi: 10.1186/1471-2334-12-186 22876748 PMC3507907

[161] Klempner MS Hu LT Evans J Schmid CH Johnson GM Trevino RP. Two controlled trials of antibiotic treatment in patients with persistent symptoms and a history of Lyme disease. N Engl J Med. (2001) 345:85–92. doi: 10.1056/NEJM200107123450202 11450676

[162] Fallon BA Keilp JG Corbera KM Petkova E Britton CB Dwyer E. A randomized, placebo-controlled trial of repeated IV antibiotic therapy for Lyme encephalopathy. Neurology. (2008) 70:992–1003. doi: 10.1212/01.WNL.0000284604.61160.2d 17928580

[163] Delong AK Blossom B Maloney EL Phillips SE. Antibiotic retreatment of Lyme disease in patients with persistent symptoms: a biostatistical review of randomized, placebo-controlled, clinical trials. Contemp Clin Trials. (2012) 33:1132–42. doi: 10.1016/j.cct.2012.08.009 22922244

[164] Kim W Zhu W Hendricks GL Van Tyne D Steele AD Keohane CE. A new class of synthetic retinoid antibiotics effective against bacterial persisters. Nature. (2018) 556:103–7. doi: 10.1038/nature26157 29590091 PMC6462414

[165] Zheng EJ Stokes JM Collins JJ. Eradicating bacterial persisters with combinations of strongly and weakly metabolism-dependent antibiotics. Cell Chem Biol. (2020) 27:1544–1552.e3. doi: 10.1016/j.chembiol.2020.08.015 32916087

[166] Wilmaerts D Windels EM Verstraeten N Michiels J. General mechanisms leading to Persister formation and awakening. Trends Genet. (2019) 35:401–11. doi: 10.1016/j.tig.2019.03.007 31036343

[167] Dewachter L Fauvart M Michiels J. Bacterial heterogeneity and antibiotic survival: understanding and combatting persistence and heteroresistance. Mol Cell. (2019) 76:255–67. doi: 10.1016/j.molcel.2019.09.028 31626749

[168] Kaldalu N Hauryliuk V Turnbull KJ La Mensa A Putrinš M Tenson T. *In vitro* studies of persister cells. Microbiol Mol Biol Rev. (2020) 84:e00070-20. doi: 10.1128/MMBR.00070-20 PMC7667008 33177189

[169] Feng J Wang T Shi W Zhang S Sullivan D Auwaerter PG. Identification of novel activity against *Borrelia burgdorferi* persisters using an FDA approved drug library. Emerg Microbes Infect. (2014) 3:e49. doi: 10.1038/emi.2014.53 26038747 PMC4126181

[170] Feng J Weitner M Shi W Zhang S Sullivan D Zhang Y. Identification of additional anti-persister activity against *Borrelia burgdorferi* from an FDA drug library. Antibiotics (Basel). (2015) 4:397–410. doi: 10.3390/antibiotics4030397 27025631 PMC4790293

[171] Feng J Shi W Zhang S Zhang Y. Identification of new compounds with high activity against stationary phase *Borrelia burgdorferi* from the NCI compound collection. Emerg Microbes Infect. (2015) 4:e31. doi: 10.1038/emi.2015.31 26954881 PMC5176177

[172] Feng J Shi W Zhang S Sullivan D Auwaerter PG Zhang Y. A drug combination screen identifies drugs active against amoxicillin-induced round bodies of *in vitro Borrelia burgdorferi* persisters from an FDA drug library. Front Microbiol. (2016) 7:743. doi: 10.3389/fmicb.2016.00743 27242757 PMC4876775

[173] Pothineni VR Wagh D Babar MM Inayathullah M Solow-Cordero D Kim KM. Identification of new drug candidates against *Borrelia burgdorferi* using high-throughput screening. Drug Des Devel Ther. (2016) 10:1307–22. doi: 10.2147/DDDT.S101486 PMC4827596 27103785

[174] Alvarez-Manzo HS Zhang Y Shi W. Evaluation of disulfiram drug combinations and identification of other more effective combinations against stationary phase *Borrelia burgdorferi*. Antibiotics (Basel). (2020) 9:542. doi: 10.3390/antibiotics9090542 32858987 PMC7559458

[175] Potula HSK Shahryari J Inayathullah M Malkovskiy AV Kim KM Rajadas J. Repurposing disulfiram (tetraethylthiuram disulfide) as a potential drug candidate against *Borrelia burgdorferi in vitro* and *in vivo*. Antibiotics (Basel). (2020) 9:633. doi: 10.3390/antibiotics9090633 32971817 PMC7557442

[176] Gao J Gong Z Montesano D Glazer E Liegner K. “Repurposing” disulfiram in the treatment of Lyme disease and babesiosis: retrospective review of first 3 years’ experience in one medical practice. Antibiotics (Basel). (2020) 9:868. doi: 10.3390/antibiotics9120868 33291557 PMC7761882

[177] Wu X Sharma B Niles S O’Connor K Schilling R Matluck N. Identifying Vancomycin as an effective antibiotic for killing *Borrelia burgdorferi*. Antimicrob Agents Chemother. (2018) 62:e01201-18. doi: 10.1128/AAC.01201-18 30126963 PMC6201113

[178] Leimer N Wu X Imai Y Morrissette M Pitt N Favre-Godal Q. A selective antibiotic for Lyme disease. Cells. (2021) 184:5405–5418.e16. doi: 10.1016/j.cell.2021.09.011 PMC8526400 34619078

[179] Pothineni VR Potula HSK Ambati A Mallajosyula VVA Sridharan B Inayathullah M. Azlocillin can be the potential drug candidate against drug-tolerant *Borrelia burgdorferi* sensu stricto JLB31. Sci Rep. (2020) 10:3798. doi: 10.1038/s41598-020-59600-4 32123189 PMC7052277

[180] Feng J Zhang S Shi W Zhang Y. Ceftriaxone pulse dosing fails to eradicate biofilm-like microcolony. Front Microbiol. (2016) 7:1744. doi: 10.3389/fmicb.2016.01744 27867375 PMC5095124

[181] Feng J Weitner M Shi W Zhang S Zhang Y. Eradication of biofilm-like microcolony structures of *Borrelia burgdorferi* by Daunomycin and Daptomycin but not Mitomycin C in combination with doxycycline and cefuroxime. Front Microbiol. (2016) 7:62. doi: 10.3389/fmicb.2016.00062 26903956 PMC4748043

[182] Horowitz RI Murali K Gaur G Freeman PR Sapi E. Effect of dapsone alone and in combination with intracellular antibiotics against the biofilm form of *B. burgdorferi*. BMC Res Notes. (2020) 13:455. doi: 10.1186/s13104-020-05298-6 32993780 PMC7523330

[183] Horowitz RI Freeman PR. Efficacy of double-dose dapsone combination therapy in the treatment of chronic Lyme disease/post-treatment Lyme disease syndrome (PTLDS) and associated co-infections: a report of three cases and retrospective chart review. Antibiotics (Basel). (2020) 9:725. doi: 10.3390/antibiotics9110725 33105645 PMC7690415

[184] Feng J Zhang S Shi W Zubcevik N Miklossy J Zhang Y. Selective essential oils from spice or culinary herbs have high activity against stationary phase and biofilm. Front Med (Lausanne). (2017) 4:169. doi: 10.3389/fmed.2017.00169 29075628 PMC5641543

[185] Feng J Shi W Miklossy J Tauxe GM McMeniman CJ Zhang Y. Identification of essential oils with strong activity against stationary phase *Borrelia burgdorferi*. Antibiotics (Basel). (2018) 7:89. doi: 10.3390/antibiotics7040089 30332754 PMC6316231

[186] Socarras KM Theophilus PAS Torres JP Gupta K Sapi E. Antimicrobial activity of bee venom and Melittin against *Borrelia burgdorferi*. Antibiotics (Basel). (2017) 6:31. doi: 10.3390/antibiotics6040031 29186026 PMC5745474

[187] Theophilus PA Victoria MJ Socarras KM Filush KR Gupta K Luecke DF. Effectiveness of *Stevia Rebaudiana* whole leaf extract against the various morphological forms of *Borrelia Burgdorferi* in vitro. Eur J Microbiol Immunol (Bp). (2015) 5:268–80. doi: 10.1556/1886.2015.00031 26716015 PMC4681354

[188] Goc A Niedzwiecki A Rath M. In vitro evaluation of antibacterial activity of phytochemicals and micronutrients against *Borrelia burgdorferi* and *Borrelia garinii*. J Appl Microbiol. (2015) 119:1561–72. doi: 10.1111/jam.12970 26457476 PMC4738477

[189] Lin YP Li L Zhang F Linhardt RJ. *Borrelia burgdorferi* glycosaminoglycan-binding proteins: a potential target for new therapeutics against Lyme disease. Microbiology. (2017) 163:1759–66. doi: 10.1099/mic.0.000571 29116038 PMC5845733

[190] Sell MG Alcorta DA Padilla AE Nollner DW Hasenkampf NR Lambert HS. Visualizing *Borrelia burgdorferi* infection using a small-molecule imaging probe. J Clin Microbiol. (2021) 59:e0231320. doi: 10.1128/jcm.02313-20 33910962 PMC8218751

[191] Jernigan DA Hart MC Dodd KK Jameson S Farney T. Induced native phage therapy for the treatment of Lyme disease and relapsing fever: a retrospective review of first 14 months in one clinic. Cureus. (2021) 13:e20014. doi: 10.7759/cureus.20014 34873551 PMC8636187

[192] Oosting M Kerstholt M Ter Horst R Li Y Deelen P Smeekens S. Functional and genomic architecture of *Borrelia burgdorferi*-induced cytokine responses in humans. Cell Host Microbe. (2016) 20:822–33. doi: 10.1016/j.chom.2016.10.006 27818078

[193] Shemenski J. Cimetidine as a novel adjunctive treatment for early stage Lyme disease. Med Hypotheses. (2016) 128:94–100. doi: 10.1016/j.mehy.2016.03.015 27107653

[194] Xu Q Seemanapalli SV Reif KE Brown CR Liang FT. Increasing the recruitment of neutrophils to the site of infection dramatically attenuates *Borrelia burgdorferi* infectivity. J Immunol. (2007) 178:5109–15. doi: 10.4049/jimmunol.178.8.5109 17404293

[195] Ritzman AM Hughes-Hanks JM Blaho VA Wax LE Mitchell WJ Brown CR. The chemokine receptor CXCR2 ligand KC (CXCL1) mediates neutrophil recruitment and is critical for development of experimental Lyme arthritis and carditis. Infect Immun. (2010) 78:4593–600. doi: 10.1128/IAI.00798-10 20823213 PMC2976349

[196] Lu RM Hwang YC Liu IJ Lee CC Tsai HZ Li HJ. Development of therapeutic antibodies for the treatment of diseases. J Biomed Sci. (2020) 27:1. doi: 10.1186/s12929-019-0592-z 31894001 PMC6939334

[197] Castelli MS McGonigle P Hornby PJ. The pharmacology and therapeutic applications of monoclonal antibodies. Pharmacol Res Perspect. (2019) 7:e00535. doi: 10.1002/prp2.535 31859459 PMC6923804

[198] Wang Y Kern A Boatright NK Schiller ZA Sadowski A Ejemel M. Pre-exposure prophylaxis with OspA-specific human monoclonal antibodies protects mice against tick transmission of Lyme disease spirochetes. J Infect Dis. (2016) 214:205–11. doi: 10.1093/infdis/jiw151 27338767 PMC4918831

[199] Wang Y Esquivel R Flingai S Schiller ZA Kern A Agarwal S. Anti-OspA DNA-encoded monoclonal antibody prevents transmission of spirochetes in tick challenge providing sterilizing immunity in mice. J Infect Dis. (2019) 219:1146–50. doi: 10.1093/infdis/jiy627 30476132 PMC6420172

[200] Schiller ZA Rudolph MJ Toomey JR Ejemel M LaRochelle A Davis SA. Blocking *Borrelia burgdorferi* transmission from infected ticks to nonhuman primates with a human monoclonal antibody. J Clin Invest. (2021) 131:e144843. doi: 10.1172/JCI144843 33914704 PMC8159683

[201] Schutzer SE Angel TE Liu T Schepmoes AA Clauss TR Adkins JN. Distinct cerebrospinal fluid proteomes differentiate post-treatment Lyme disease from chronic fatigue syndrome. PLoS One. (2011) 6:e17287. doi: 10.1371/journal.pone.0017287 21383843 PMC3044169

[202] Nilsson K Skoog E Jones V Labbé Sandelin L Björling C Fridenström E. A comprehensive clinical and laboratory evaluation of 224 patients with persistent symptoms attributed to presumed tick-bite exposure. PLoS One. (2021) 16:e0247384. doi: 10.1371/journal.pone.0247384 33735220 PMC7971513

[203] Nilsson K Skoog E Edvinsson M Mårtensson A Olsen B. Protein biomarker profiles in serum and CSF in 158 patients with PTLDS or persistent symptoms after presumed tick-bite exposure compared to those in patients with confirmed acute neuroborreliosis. PLoS One. (2022) 17:e0276407. doi: 10.1371/journal.pone.0276407 36327322 PMC9632922

[204] Hein TM Sander P Giryes A Reinhardt JO Hoegel J Schneider EM. Cytokine expression patterns and single nucleotide polymorphisms (SNPs) in patients with chronic borreliosis. Antibiotics (Basel). (2019) 8:107. doi: 10.3390/antibiotics8030107 31366164 PMC6784230

[205] Tokarska-Rodak M Fota-Markowska H Pańczuk A Matuska K Zarębska M. Analysis of the concentration of selected serological parameters in patients undergoing antibiotic treatment of Lyme disease and assessment of their potential application in the control of the therapy effectiveness - pilot study. Ann Agric Environ Med. (2021) 28:605–11. doi: 10.26444/aaem/132786 34969217

[206] Morrissette M Pitt N González A Strandwitz P Caboni M Rebman AW. A distinct microbiome signature in Posttreatment Lyme disease patients. mBio. (2020) 11:e02310-20. doi: 10.1128/mBio.02310-20 32994327 PMC7527730

[207] Fitzgerald BL Molins CR Islam MN Graham B Hove PR Wormser GP. Host metabolic response in early Lyme disease. J Proteome Res. (2020) 19:610–23. doi: 10.1021/acs.jproteome.9b00470 31821002 PMC7262776

[208] Fitzgerald BL Graham B Delorey MJ Pegalajar-Jurado A Islam MN Wormser GP. Metabolic response in patients with post-treatment Lyme disease symptoms/syndrome. Clin Infect Dis. (2020) 73:e2342–9. doi: 10.1093/cid/ciaa1455 PMC8492154 32975577

